# On the Application of Entropy-Based Metrics for UltraWideBand Line of Sight (LOS)/Not LOS (NLOS) Classification with Ensemble Instance Selection

**DOI:** 10.3390/e28080904

**Published:** 2026-08-12

**Authors:** Gianmarco Baldini

**Affiliations:** European Commission, Joint Research Centre, 21027 Ispra, Italy; gianmarco.baldini@ec.europa.eu

**Keywords:** UltraWideBand, machine learning, entropy

## Abstract

The knowledge of the Line of Sight (LOS) or Not Line of Sight (NLOS) propagation condition is useful information in wireless communication system. Such knowledge can be inferred by the analysis of the signal, by using specific signal structures (e.g., preambles) or by the application of machine learning (ML) algorithms. In recent times, deep learning (DL) has been applied with success to the classification of LOS/NLOS conditions but with a significant computational time, which can be a practical issue in computing constrained devices. On the other hand, ML relies on the identification of key discriminating features, which can enhance the classification performance. This paper explores the application of entropy metrics to this classification problem. Beyond Shannon entropy, researchers have developed various entropy metrics in recent years in various domains (e.g., healthcare), but they have been scarcely applied to UWB LOS/NLOS classification to the best of the author’s knowledge. This paper addresses this gap by applying entropy metrics in combination with ML classifiers to the public eWINE dataset, characterised by seven different propagation environments where UWB signals were transmitted and recorded in LOS and NLOS conditions. The results presented in this paper show that entropy metrics can significantly enhance the LOS/NLOS classification accuracy and can produce an overall competitive performance. In addition, this paper presents a novel instance selection approach based on the use of entropy metrics, which is demonstrated to significantly outperform even the direct application of some DL algorithms on the basis of the results presented in the literature on the same eWine data set. To summarise the novelty aspects of this study, for the first time in the literature, this study presents an extensive analysis of the discriminative advantage (discrimination index) of entropy measures introduced in the research literature in other domains (e.g., mechanical problems, analysis of physiological signals) in UWB multipath environments for UWB LOS/NLOS classification. In addition, this study presents for the first time the application of an ensemble instance selection algorithm based on entropy measures to the problem of UWB LOS/NLOS classification to handle “noise” or “boundary” samples in the data set, thereby improving model generalisation.

## 1. Introduction

The communication performance of wireless services is significantly affected by the type of propagation channel [1] and if the transmitted signal and receiver are in Line Of Sight (LOS) or Not Line of Sight (NLOS) conditions. For example, in NLOS conditions, the transmitted signal can be negatively impacted by various attenuation and fading effects. As a consequence, knowledge of the propagation channel and especially the LOS/NLOS condition can be useful to enhance the quality of the communication system because it can adopt measures to counteract negative effects such as increasing the transmitter power.

The focus of this paper is on UltraWideBand (UWB) signals. UWB is a radio technology that can use a very low energy level for short-range, high-bandwidth communications over a large portion of the radio spectrum [2].

Knowledge of the LOS/NLOS condition is a binary classification problem, which can be solved using statistical means using the Probability Density Function (PDF) of the received signal strength, the K-factor in the Rician fading model [3] or the Root Mean Square (RMS) delay spread or the mean access delay in [4]. More recently, machine learning (ML) (including deep learning (DL)) algorithms have been adopted to identify the type of propagation channel. For example, the authors in [1,5,6] have used support vector machine (SVM), Random Forest (RF), K nearest neighbor (KNN) algorithms to classify LOS/NLOS conditions using hand-crafted features such as skewness, kurtosis, standard deviation while other authors have exploited DL algorithms such as [7] where a convolutional neural network (CNN) or a sophisticated ADCNN-BiLSTM transformer architecture was used in [8] at a very significant computing cost, especially in the training phase. Additional details on the literature on UWB LOS/NLOS classification are provided in Section 2.

There is a trade-off in the design of the channel estimator because the computational time must be minimised on one side, while the channel classification accuracy has to be maximised on the other side as described in surveys like [5]. Taking into consideration that the LOS/NLOS propagation condition has to be identified within a very short time and that the channel propagation conditions may change frequently in moving receivers, which may require re-training of the ML model, it is imperative to look for LOS/NLOS classification techniques, which may still be accurate to a certain degree while minimising both the training and inference times. On the other hand, LOS/NLOS classification using computing efficient ML approaches based on hand-crafted features is heavily dependent on the choice of the features themselves. Based on recent results in which entropy measures were successfully used in the UWB LOS/NLOS classification in combination with the time series classification algorithm [9], this paper proposes an evaluation of the application of the entropy measure as classification features, especially with the view that several new definitions of entropy have been proposed in the literature in domains other than wireless communication (e.g., healthcare, finance, physiological signals). The main underlying concept of this paper is to investigate how suitable these measures are for the specific LOS/NLOS classification problem.

In addition, this paper presents a novel instance selection algorithm based on the parallel application of the most discriminating features identified by a feature selection algorithm of both filter- or wrapper type. The application of such an instance selection algorithm on the eWine data set [10] is shown to outperform results reported in the literature, even with the application of DL algorithms such as CNN and Long Short-Term Memory (LSTM).

The key novelty aspects of this study are summarised here:

- for the first time in the literature, this study presents an extensive analysis of the discriminative advantage (discrimination index) of entropy measures in UWB multipath environments for UWB LOS/NLOS classification.
- Entropy measures, which have been recently introduced in the research literature in other domains (e.g., mechanical problems, analysis of physiological signals) have been applied for the first time in the wireless communication domain and more specifically to the problem of UWB propagation environment classification.
- For the first time, an ensemble instance selection algorithm based on entropy measures has been applied to UWB LOS/NLOS classification to handle “noise” or “boundary” samples in the data set, thereby improving model generalisation.

The structure of the paper is as follows. Section 2 provides an overview of the research literature on the classification of LOS/NLOS of UWB integrating what was already presented in this section. Section 3 describes the general methodology of the proposed approach, including the evaluation metrics, the adopted ML algorithms, and the computing platform. Section 3 also provides background information on UWB technology and signal. Section 4 describes the experimental setting used in the study with the hardware and software platform. Section 5 describes the data set used to evaluate the approach. The entropy features are described in Section 6 while the non-entropy features are described in Section 7. Section 8 presents the results of the proposed approach based on entropy metrics and other features and compares them with the results obtained with CNN, LSTM and from the literature. Finally, Section 9 provides a summary of the key conclusions of the study, its main limitations, and outlines future developments.

## 2. Related Work

This section aims to provide an overview on the research literature on the LOS/NLOS classification problem in wireless communication and, more specifically, on UWB LOS/NLOS classification.

As described previously in Section 1, knowledge of the LOS/NLOS condition is a binary classification problem. Channel information can be extracted from the signal in space by using hand-crafted features or by using DL algorithms where the features are created by the algorithm itself. On the other hand, DL algorithms require significant time for training and inference. In addition, the generated features are often not explainable, which is a common problem in the application of DL [11]. For the sake of completeness, it can be mentioned that there are also other potential approaches which can be used for LOS/NLOS classification. One would be to apply statistical frameworks [12], which model the complete sampled trajectory (i.e., the Channel Impulse Response (CIR)) as high dimensional data directly [13] rather than summarising it through handcrafted scalar features as in this study. This is based on the consideration that CIR observations are densely sampled signals with strong dependence across neighbouring sampling locations, and functional data methods provide an alternative framework for preserving this signal structure rather than reducing each CIR entirely to scalar summaries. In wireless communication, functional data methods were used in [14] for Wi-Fi sensing but not for UWB classification. Another approach would be to apply time series classification algorithms like the Supervised Time Series Forest used in [9].

Historically, hand-crafted features were preferred for this type of classification problem. In particular, statistical means using the PDF of the signal strength received, the K-factor in the Rician fading model were used in [3], while the RMS delay spread or the mean access delay was used in [4]. In the UWB LOS/NLOS classification, the authors of [15] have used the standard deviation of noise, the maximum noise value, and the first path amplitude values together with KNN. In [16], the author used variance, skewness, kurtosis, Shannon entropy, quantile with a threshold of 0.7, and quantile with a threshold of 0.9.

Other examples of the application of hand-crafted features in combination with non-DL ML algorithms are presented in [1,5,6], where SVM, RF, KNN algorithms were used to classify LOS/NLOS conditions using hand-crafted features such as skewness, kurtosis, standard deviation.

Entropy features were also used for LOS/NLOS classification in a few studies, where they have demonstrated significant discriminant power, which was the rationale for adopting them in this paper. In particular, the authors of [9] have enhanced the Supervised Time Series Forest classification algorithm with entropy features for LOS/NLOS classification. In another study, the authors of [17] have shown that the Shannon entropy metric has a higher detection/identification rate compared to RMS delay spread and kurtosis. This study used a much larger set of entropy metrics than those adopted in previous papers.

As mentioned above, recent studies have adopted DL algorithms and architectures for the classification of LOS/NLOS and, more specifically, for UWB.

In [7], the authors have applied a combination of CNN and LSTM algorithms to the same eWine data set used in this study in different configurations. In particular, CNN+stacking LSTM manages to obtain an average classification accuracy of 82.14% outperforming both CNN and CNN-LSTM. Another study [18] applied de-noising techniques in combination with a CNN architecture to the same problem and the same data set, obtaining an accuracy of 81.41%.

Spiking Neural Networks (SNN) were used in the eWine data set in [19], where it obtained a classification accuracy of 81.8%. Even if it is slightly lower than the results in [7], the computing power for SNN is much lower than that of CNN+stacking LSTM.

A sophisticated ADCNN-BiLSTM transformer architecture was used in [8] at a very significant computing cost, especially in the training phase, obtaining a state-of-the-art classification accuracy of 92.19%.

Another DL architecture focused on improving the classification of UWB LOS/NLOS was proposed in [20], with a resource-efficient DL method for UWB NLOS Identification based on selective state-space modelling obtaining a classification precision between 86% and 87%.

However, none of the proposed approaches presented in the literature have adopted an instance selection algorithm to improve classification accuracy. In addition, very few studies have used a large set of entropy features as in this study and relied only on the Shannon entropy, while more sophisticated entropy features could be used.

## 3. Methodology

### 3.1. Workflow

**Figure 1:**
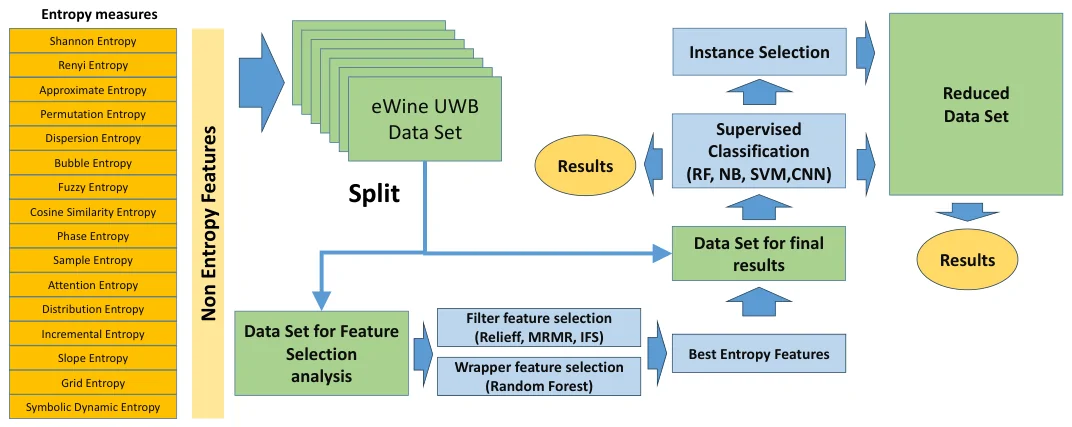
Set of procedures of the proposed approach (color code: blue for processes, green for data sets).

The workflow of the proposed approach is relatively simple and is described in detail in Figure 1. The workflow is composed of three main phases. The first phase is used to normalise the data and identify the set of most discriminating entropy features. In the second phase, the entropy features determined in the previous step are used to estimate the accuracy of the classification. In the third phase, a novel instance selection approach based on the hard label outputs of the specific entropy measures is used to select specific instances used to reduce the data set.

Each step of the different phases is described in the following paragraphs. In the first phase, the eWine data set [10] is normalised using the RMS of each CIR. To minimise the application of the entropy measures, the most relevant part of the UWB pulse of size 512 was selected. There are 6000 samples of UWB CIR in each of the seven environments in the eWine data set, as described in detail in Section 5. For each propagation environment, one part of the data set composed of 1000 samples (randomly selected among the 6000 samples) was selected for the feature selection phase. This is the data set for the analysis of the selection of features shown in Figure 1 and is called $DS_{FS}$ in the rest of this paper. The rest of the data set is shown as the data set for the final results in Figure 1 and is called $DS_{RS}$ in this article. More accurately, there is one $DS_{FS_{i}}$ portion of the data set for feature selection and one $DS_{RS_{i}}$ portion for the production of the final results, where *i* = 1, …, 7 identifies the propagation environment.

The author would like to highlight the following:

1. For each propagation environment *i*, $DS_{FS_{i}}$ and $DS_{RS_{i}}$ are cleanly separated and no data from $DS_{RS_{i}}$ is used in $DS_{FS_{i}}$.
2. For each propagation environment *i*, 1000 samples are identified in a random way from the total of 6000 samples to produce $DS_{FS_{i}}$. In this process, the data sets of each propagation environment were kept distinct from the others.
3. To mitigate the risk of overfitting, the classification is implemented using a 4-fold approach separately in $DS_{FS_{i}}$ and $DS_{RS_{i}}$. In the 4-fold approach, 3/4 of the data set is used for training and 1/4 is used for testing. The final results of the performance metrics (e.g., accuracy) are the average across the 4 executions of the ML algorithm.

Various entropy measures are applied to the entire UWB data set in addition to the non-entropy measure identified already in the research literature. The list of features is described in detail in Section 6 for entropy measures and Section 7 for non-entropy measures.

The application of features generates feature spaces both from $DS_{FS}$ and $DS_{RS}$, which are, respectively, $FS_{FS}$ and $FS_{RS}$. Both filter and wrapper FSAs are applied to the $FS_{FS}$ feature space to evaluate the performance of entropy-based features.

The Feature Selection Algorithm (FSA) can be divided into wrapper methods where the features are identified in combination with a classifier and the filter methods, which rely on data analysis without the use of a classifier. Both methods are used in this article with the RF classifier used in the wrapper methods. The filter FSAs is described in Section 3.4.

The FSAs are mostly used in this study to select the entropy features, which are then combined with the non-entropy features also used in this study.

In the second phase, the combinations of the best selected entropy features with the non-entropy features are used in the $DS_{RS}$ portion of the data set to estimate the classification accuracy. The combinations are generated on the basis of the following criteria: (1) for the filter FSA, we select the highest ranking entropy features identified by the FSA with a cumulative weight of 95% on the overall sum of feature weights provided by the FSA. (2) for the wrapper FSAs, we select the best 5, 10, 15 entropy features and the set of entropy features, which have a higher classification accuracy than the non-entropy features on the $DS_{FS}$ portion of the data set (the latter is called BA in the rest of this paper). For the wrapper FSAs, we selected this approach because of its computing speed in comparison to the application of sequential feature selection methods, which may be quite time-consuming.

**Figure 2:**
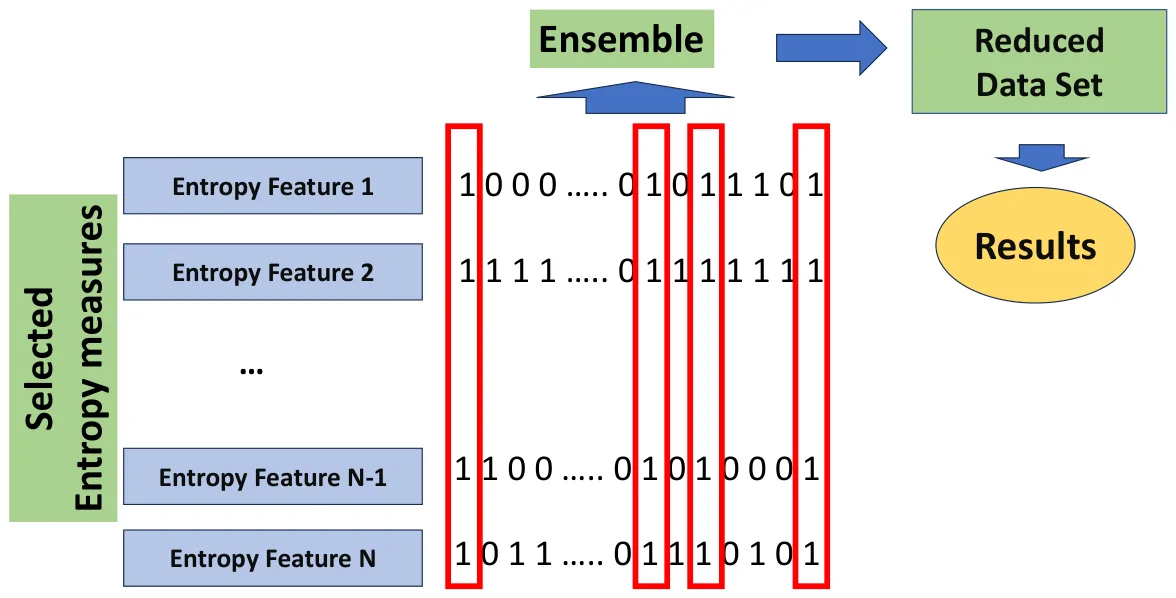
Pictorial description of the instance selection method.

The third and final phase implement the instance selection algorithm using the ensemble of classification results from the features selected in the second phase. In such a phase, each entropy measure combined with the non-entropy measure is used to determine the predicted hard label information. In this context, the hard label denotes the specific predicted value of the LOS or NLOS conditions in opposition to the probability that an instance (or sample) is predicted to be LOS or NLOS. The hard label information is combined from each predictor based on the specific entropy measure with a simple rule of unanimity: if all the predictors agree on a specific label information (either LOS or NLOS), then that sample is selected. A pictorial description of the unanimous rule for instance selection is shown in Figure 2. The red boxes delimit the hard labels identified by all the classifiers based on the specific entropy metrics.

There are many ensemble strategies in the literature including unanimous voting, majority voting, probability-based voting, etc. [21]. An extensive analysis of the different voting schemes is beyond the scope of this paper. In the various ensemble voting schemes, the majority vote (where unanimous voting belongs) is distinguished for its simplicity and efficiency [22]. The unanimous vote where all voting participants must agree on specific labels is quite frequently used in the literature [22,23]. In this specific context, it tends to minimise the number of returned samples (thus lowering the computing complexity), which is one of the goals of the approach considering the large number of samples in each propagation environment (i.e., 6000 samples). The additional advantage of unanimous voting is its simplicity as it is not known a priori which majority of entropy features is needed to produce the best results, and such an optimisation step should be repeated in this context for all the propagation environments. On the other hand, unanimous voting is reported to be more effective in filtering false positives, with the false negatives rate being increased [23]. The application of more sophisticated ensemble voting schemes is deferred to future developments of this study.

An algorithm description of the flow described above is presented in Algorithm 1. In the algorithm:

- NENV is the number of propagation environments in the data set (i.e., 7);
- $OptFeat_{i}$ is the set of best features obtained by the filter or wrapper method on the feature space $DS_{FS_{i}}$. The set has a dimension $ONF_{i}$, which may be different for each filter/wrapper FSA and environment *i*;
- $HL_{i}^{j}$ is the set of hard labels obtained by the application of the classifier for each environment i and entropy feature *j*;
- $ISLabel_{i}$ is the set of labels identified with the unanimous ensemble instance selection step described in Figure 2;
- $Acc_{i}$,$Rec_{i}$,$Pre_{i}$ are the final performance metrics obtained for the propagation environment *i*.

**Algorithm 1** High Level algorithmic description of the proposed workflow**for** i←1,NENV **do**    Split Data set in $DS_{FS_{i}}$ and $DS_{RS_{i}}$.    Apply feature extraction to $DS_{FS_{i}}$ to produce $FS_{FS_{i}}$.    Apply feature extraction to $DS_{RS_{i}}$ to produce $FS_{RS_{i}}$.    $OptFeat_{i}$ (J=1, …, $ONF_{i}$) ← Feature selection (filter and wrapper) applied to $DS_{FS_{i}}$    **for** j ← 1, $ONF_{i}$ **do**        $HL_{i}^{j}$ ← Application of classifier using $OptFeat_{i}^{j}$ on $DS_{RS_{i}}$        $ISLabel_{i}$ ← Unanimous ensemble of $HL_{i}^{j}$        $Acc_{i}$,$Rec_{i}$,$Pre_{i}$ ← Application of classifier using $OptFeat_{i}^{j}$ on $DS_{RS_{i}}$ with $ISLabel_{i}$    **end for****end for**

This process may seem to generate a low number of selected instances, but the empirical results shown in Section 8 show that there is good agreement among the predictors and the number of selected instances is relatively high compared to the entire data set.

Finally, different classifiers (Naive Bayes, SVM, RF) are applied to the reduced data set. The results are compared with the results presented in the literature on the eWine data set.

### 3.2. Background Information on UltraWideBand Technology

UltraWideband (UWB) is a radio technology, which can use a large portion of the radio spectrum with low energy level for short-range, high-bandwidth communications. It is used in a variety of applications including communications, precise location, and tracking. The Federal Communications Commission in USA (FCC) has defined an UWB device as any device with a −10 dB fractional bandwidth, greater than 20% or occupying at least 500 MHz of the spectrum [24]. Most narrowband systems occupy less than 10% of the centre frequency bandwidth, and are transmitted at much higher power levels [25]. The performance of the UWB technology can be hampered by the presence of obstacles and reflecting surfaces, which are common in indoor environments. As a consequence, the knowledge of LOS or NLOS conditions is useful for the transmitter and receiver components to implement mitigation measures.

UWB can be used to characterise an indoor propagation environment. This technique is based on employing a UWB narrow pulse to probe the channel. The measurement resolution is equal to the width of transmitted pulse [26].

The UWB channel can be described by its time-variant impulse response or CIR, which can be expressed in the following equation as:

(1) $$h\left(t,\tau \right)=\sum_{n=1}^{N(t)}a_{n}\left(t\right)\delta (t-\tau_{n}\left(t\right))e^{j\theta_{n}\left(t\right)}$$

where the parameters of the *N*th path, $a_{n}$,$\tau_{n}$ and $\theta_{n}$ are the amplitude, delay, phase, and number of relevant multipath components, respectively [26]. Equation (1) also provides insight into why the entropy measures described in Section 6 can have a discriminating power for the UWB LOS/NLOS classification. The values of $a_{n}$, $\tau_{n}$ and $\theta_{n}$ change significantly between LOS and NLOS propagation environments, creating not only different distributions of values in the received impulse response (which can be exploited by the Shannon and Renyi entropies measures), but also in the temporal relation or order among the values (which can be exploited by permutation entropy measures or bubble entropy measures). The sequence of amplitude values in the CIR can be different among the various propagation environments because obstacles may change in different ways the amplitude or delays. As a consequence, some entropy measures may have more discriminating power than others in specific propagation environments. In addition to entropy measures, classical features such as mean, variance, skewness, or kurtosis can also provide relevant information, which is the reason why they are also used in this study as described in Section 7.

### 3.3. Machine Learning Algorithms

Three different classifiers are used in different phases of the proposed approach. The main machine learning algorithm used in the first and second phases is the RF algorithm, which was chosen for its scalability and computing efficiency. The Random Forest was implemented using the function *fitcensemble* of MATLAB Release 2016b with the AdaBoost M1 algorithm and a maximum number of splits for the decision trees equal to 12. The Gini index was used as a split criterion. These values were chosen as the result of a grid approach in which the best values were chosen averaging the grid results of the filter and wrapper methods in the $FS_{F}S$ portion of the data set.

The other classifier algorithms used in the third phase to evaluate the classification performance of the set of entropy features selected in the previous phases are the Naive Bayes algorithm and the SVM. Both are implemented using the *fitecoc* MATLAB function and different templates. In particular, the SVM template is defined with the Radial Basis Function (RBF) kernel with $kernelscale=2^{6}$ and $Cfactor=2^{8}$. As in the random forest, the values for SVM were chosen as the result of a grid approach where the best values were chosen averaging the grid outcomes from the filter and wrapper methods in the $FS_{F}S$ portion of the data set.

For all classifications, the results presented are generated by averaging the results of 10 executions, using a K-fold with K=4 (i.e., 4 partitions) for a total of 40 executions of the classification algorithm.

### 3.4. Feature Selection Algorithms

In this study, we have used three different feature selection algorithms: two classical ones: ReliefF and Minimum Redundancy Maximum Relevance (mRMR) and the recently introduced Infinite Feature Selection (IFS). The algorithms were chosen for historical reasons, their scalability, and their availability in MATLAB. A description of the algorithms is provided in the following sub-sections.

#### 3.4.1. ReliefF

ReliefF is a FSA proposed by the authors of [27] and it is an improvement of the Relief algorithm [28].

The ReliefF algorithm penalises predictors that give different values to neighbours of the same class and rewards predictors that give different values to neighbours of different classes. The Relief algorithm was originally designed for binary classification problems, which is the goal of this study (LOS or NLOS condition). The *relieff* MATLAB function was used to implement the ReliefF algorithm using k = 10 nearest neighbours per class.

#### 3.4.2. Minimum Redundancy Maximum Relevance

mRMR was initially proposed in [29] and then improved in a more complete form in [30], where it is described as being based on the concept of selecting good features according to the maximal statistical dependency criterion based on mutual information. Due to the difficulty in directly implementing the maximal dependency condition, the authors first derive an equivalent form, called mRMR, to select incremental features of first-order.

The advantage of mRMR is its incremental selection scheme, which avoids the difficult multivariate density estimation to maximise the dependency.

The MATLAB function *fscmrmr* with default settings was used to implement mRMR.

#### 3.4.3. Infinite Feature Selection

The IFS was introduced by the authors of [31] in 2020. It belongs to the family of filters FSAs, which considers subsets of features as paths in a graph, where a node is a feature, and an edge indicates pairwise (customisable) relations among features, dealing with relevance and redundancy principles. The IFS algorithm is implemented in three phases. In the first phase, an undirected fully-connected weighted graph is built, where node $v_{i}$, 1 ≤ *i* ≤*n* corresponds to the feature $f_{i}$ and each edge, which connects $v_{i}$ to $v_{j}$ has weight or values, which models the expectation that features $f_{i}$ and $f_{j}$ are relevant and not redundant. In the second phase, the weighted adjacency matrix associated with the graph is used to assess the value of each feature $f_{i}$ (i.e., the i-node in the graph) while considering possible subsets of features, as they are paths of variable length. The key aspect is that the probability (which gives the reason for the name IFS) of having a particular feature in a subset of any length is calculated by summing all the possible lengths until infinite (which gives the reason for the name IFS). In the third phase of the algorithm, a threshold over the ranking is automatically selected by clustering over the ranked value. The rationale for this third phase is to identify the least two distributions, one which contains the features to keep with higher value, and the other containing the features to discard. Additional details are presented in [32]. In this FSA, the term infinite is due to the consideration that paths are allowed to be infinite to improve the computational complexity of the selection process.

The author adopted this FSA because it is highly scalable for large data sets (as in this case with 6000 samples for each propagation environment). The computing complexity of IFS is $O(n^{3}\times T)$, where T is the number of samples and n is the number of initial features. Then, IFS is linear with the number of samples and is therefore quite scalable. This study has used the MATLAB implementation of IFS provided by the authors of [33].

### 3.5. Evaluation Metrics

To evaluate the performance of the approach, we used the following metrics: accuracy, precision, and recall.

Accuracy is defined as:

(2) $$Accuracy=\frac{TP+TN}{\left(TP+FP+FN+TN\right)}$$

Recall is defined as

(3) $$Recall=\frac{TP}{\left(TP+FN\right)}$$

Precision is defined as

(4) $$Precision=\frac{TP}{\left(TP+FP\right)}$$

where TP is the number of True Positives, FP is the number of False Positives, FN is the number of False Negatives, and TN is the number of True Negatives.

## 4. Experimental Setting

### 4.1. Software Environment

The scientific computing environment uses the MATLAB Release 2024b scientific programming language from MathWorks based in Natick, Massachusetts, USA. The Random Forest (RF), Support Vector Machine (SVM) and Naive Bayes (NB) algorithms are from the Machine Learning toolbox. Other open-source libraries are used to implement the entropy measures as described in Section 6 and the FSAs described in Section 3.

As described above, a 4-fold approach was used to split the samples in both $FS_{FS}$ and $FS_{RS}$ and the results were created by averaging the result (e.g., precision) from each fold.

As described above, the approach was applied independently for each of the seven propagation environments and, in each propagation environment, the analysis and processing of the data between $DS_{FS}$ and $DS_{RS}$ (and consequently $FS_{RS}$ $FS_{FS}$) were kept separated.

### 4.2. Hardware Environment

The computing platform is an Intel I9-990KF Central Processing Unit (CPU) with a clock speed of 3.6 GHz and 32 Gbytes of Random Access Memory (RAM) and equipped with a CUDA enabled NVIDIA QUADRO RTX 4000.

## 5. Data Set

**Figure 3:**
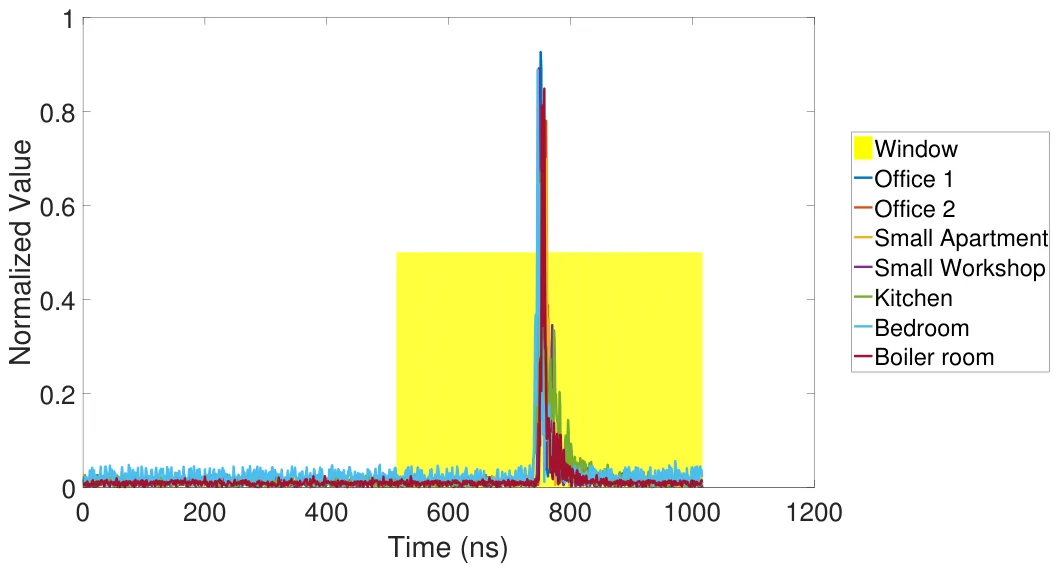
Ultrawideband Channel Impulse Response (CIR) samples from each of the seven environments and the window used to capture the segment of the CIR for feature extraction.

The eWINE data set from [10] is used to evaluate the proposed approach. The data is publicly available at https://github.com/ewine-project/UWB-LOS-NLOS-Data-Set, accessed on 16 July 2026. The data set was chosen because it was recently adopted by other researchers for the classification ML of the LOS/NLOS channel conditions [7,34]. It has a rich set of samples (6000) for each of the seven scenarios included in the data set: Office 1, Office 2, a small apartment, a small workshop, a kitchen with a living room, bedroom, and a boiler room. The data set is created using the SNPN-UWB board with DecaWave DWM1000 UWB radio module (DecaWave has now been acquired by QORVO) by transmitting UWB pulses in the seven mentioned scenarios. In each scenario, approximately 3000 LOS samples and 3000 NLOS samples were taken, using a DecaWave DWM1000 UWB radio module as an anchor and a second as a tag. In this study only data from CIR were used and no other information was used, as in [7], making it a pure classification problem from ML. Each sample is a CIR of length 1024. It was noted that only a portion of the CIR of size 512 was useful for the analysis, and subsegments of that size in the data set were used in the analysis. This can be seen in Figure 3, which shows the segment used in the analysis and samples from each of the seven environments. The signals above the noise background are practically included in the selected window.

As mentioned before, one part of the data set (called $DS_{FS}$) composed by 1000 samples (randomly selected among the 6000 samples) was selected for the feature selection phase.

The rest of the data set is called $DS_{RS}$ in the rest of this paper. It is used to produce the final results once the optimal features have been selected.

## 6. Entropy Measures

Various entropy metrics were used in this paper. Beyond the classical Shannon entropy and its variants like the Renyi entropy, a number of entropy measures recently proposed in the research literature were also used. In many cases, these entropy measures were introduced by the research community in other domains different from wireless communication (e.g., medical field and analysis of physiological signals) but are suitable for the analysis of other signals such as the UWB CIR considered in this study, as shown in Section 8. Beyond the well-known Shannon entropy and its extension, the Renyi entropy, some recent entropy measures are based on relatively new concepts. For example, the bubble entropy proposed in [35] is based on the application of the bubble sort algorithm to order a time series (e.g., the CIR in this case). Then, the bubble entropy measure is calculated as the number of swaps performed for each vector to implement the ordering. The bubble entropy is in turn based on the permutation entropy, which estimates the number of permutations in an embedding vector in the time series. Instead, the attention entropy proposed in [36] is based on the concept of analysing the frequency distribution of the intervals between the key observations in a time-series. This is particularly valuable in fading conditions analysis because the fading conditions are known to impact the frequency distribution, and this entropy measure is used to take advantage of this aspect. Two relevant parameters in the definition of most entropy measures are the embedding parameter m and the delay τ. The m parameter is the size of the embedding vectors, which are sub-sequences in the time series on which some entropy measures are calculated. For example, in the permutation entropy, with m = 3 they are the possible permutations of vectors of size 3 in the time series. There is a trade-off in the value of m. A value of m, which is too large, significantly increases the computing cost of the entropy estimate, and a value of m, which is too low, may not provide enough discriminating value. For most of the entropy measures considered in this study, it was decided to use a value of m=3 but with the choice that values of m=1 and m=2 were also estimated when applicable. The τ parameter instead defines the delay between samples in the time series. A value of τ greater than 1 may make the computation more efficient because less time series samples are used, but at the risk of losing valuable information (i.e., the not considered samples when τ is equal to or greater than 1). For this reason, the value of τ=1 was chosen. An empirical evaluation for a specific propagation scenario and different values of *m* is provided in Section 8. The complete list of the entropy measures adopted is identified and described in Table 1, Table 2 and Table 3.

This study has used the MATLAB implementation of the entropy measures available at https://ch.mathworks.com/matlabcentral/fileexchange/94185-entropyhub, accessed on 16 July 2026.

The entropy features identified above may be correlated with each other because they are defined on similar concepts or because they are just different for a value of a parameter (e.g., Renyi coefficient or the embedding factor m). This level of correlation may be slightly different in the different propagation environments.

The figures below show the linear dependence among the 37 entropy features in two examples among the seven different propagation environments. For reasons of space, not all propagation environments are shown.

The linear dependence was estimated using the *corrcoef* MATLAB function, which implements the Pearson correlation. The degree of correlation varies from 0 to 1 with 1, which indicates the maximum level of correlation. The diagonal values in the matrix in the figures are equal to 1 because they are related to the feature correlated to itself.

**Figure 4:**
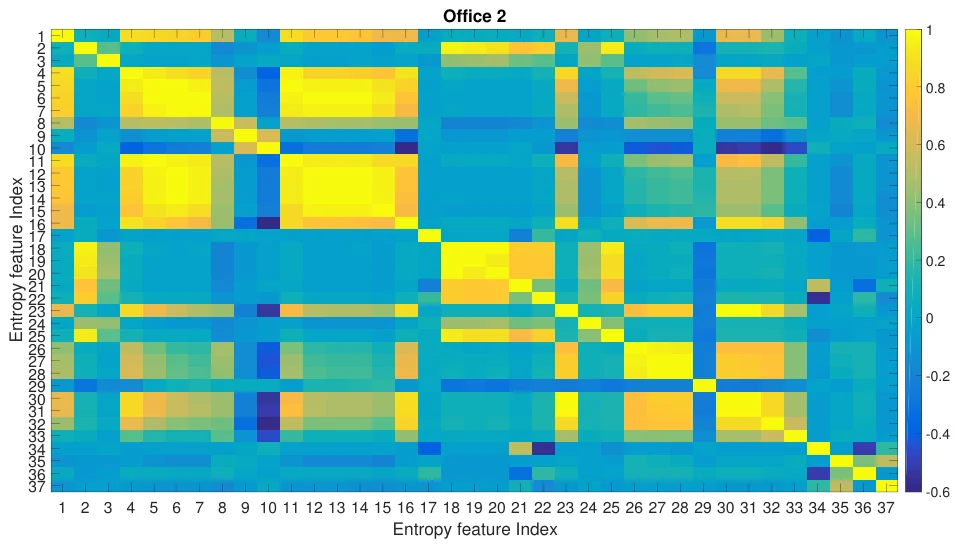
Pearson correlation matrix among the entropy features in the Office 2 environment.

**Figure 5:**
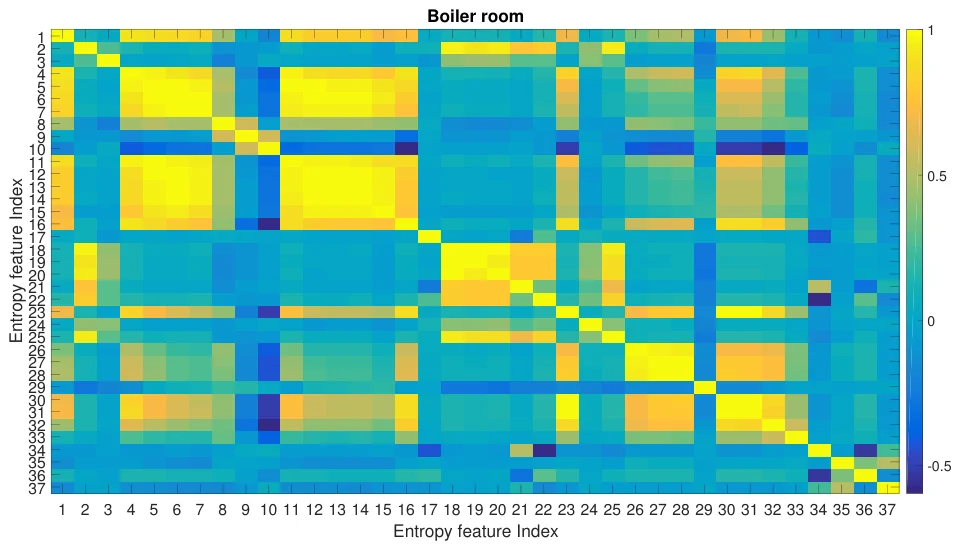
Pearson correlation matrix among the entropy features in the Boiler room environment.

Figure 4 shows the correlation of the entropy features in the Office 2 environment, while Figure 5 shows the correlation of the entropy features in the the boiler room environment. Three aspects can be extrapolated from the Figures. The first aspect is that entropy features related to the same entropy measure (feature IDs 11, 12, 13, 14 from the sample entropy) are obviously highly correlated because they are related to the same algorithm. The second aspect is that the entropy measures are generally not correlated among themselves, which improves the generalisability of the approach. The third aspect is that the Pearson correlation coefficients are usually quite similar across propagation environments. There are still some numeric differences that may not be evident in a visual inspection of the figures, but they are present.

The non-entropy features are instead described in the following Section 7 and Table 4.

## 7. Non-Entropy Features

Beyond the entropy metrics defined in Section 6, several other features not related to entropy are also defined and described in this section. These features (e.g., variance, skewness, kurtosis) are derived from similar studies in the literature [1,5,6], but are further enhanced by studies, which have shown that the number of statistical features [53] defined in different domains (e.g., healthcare, physiological signals, brain related analysis) can have a wider application than the domains where they were originally generated. The list of features is presented in Table 4.

## 8. Results

This section presents results on the application of the proposed approach on the eWine data set described in Section 5.

This section is structured to provide the results for the different phases of the approach and taking into account that both the filter and the wrapper FSAs were used. In detail, for the first and second phases of the approach, the Section 8.1 provides the result for the application of the wrapper feature selection using RF. Section 8.2 provides the results on the application of the filter feature selection algorithms (i.e., mRMR, IFS and ReliefF).

The third phase is the application of the instance selection algorithm, which can also be based on the filter or wrapper FSA. In detail, Section 8.3 provides the results based on the wrapper FSA, while Section 8.4 shows the results obtained with instance selection based on filter FSA.

This section also presents an empirical evaluation of the impact of the hyper-parameters of the classifiers and the parameter *m* in Section 8.6.

Robustness to noise is briefly evaluated in Section 8.7, where Additive White Gaussian Noise (AWGN) was added to the UWB CIR samples to evaluate the impact of noise on the classification accuracy.

Finally, an analysis of the computation time requested by the steps in each phase is provided in Section 8.8.

Depending on the phase, the results are calculated in portions of the data set $DS_{FS}$ or $DS_{RS}$.

As mentioned above, the results presented are generated by averaging the results of 10 executions, using a K-fold with K=4 (i.e., 4 partitions) for a total of 40 executions of the classification algorithm.

Note that each propagation environment may identify different sets of features. As described before, the characteristics of each propagation environment are better captured by some entropy measures than others. For example, the bubble entropy design supports discriminating aspects related to the order of the values, while the slope entropy may capture better patterns related to the slope of CIR.

### 8.1. Wrapper Feature Selection Results

**Figure 6:**
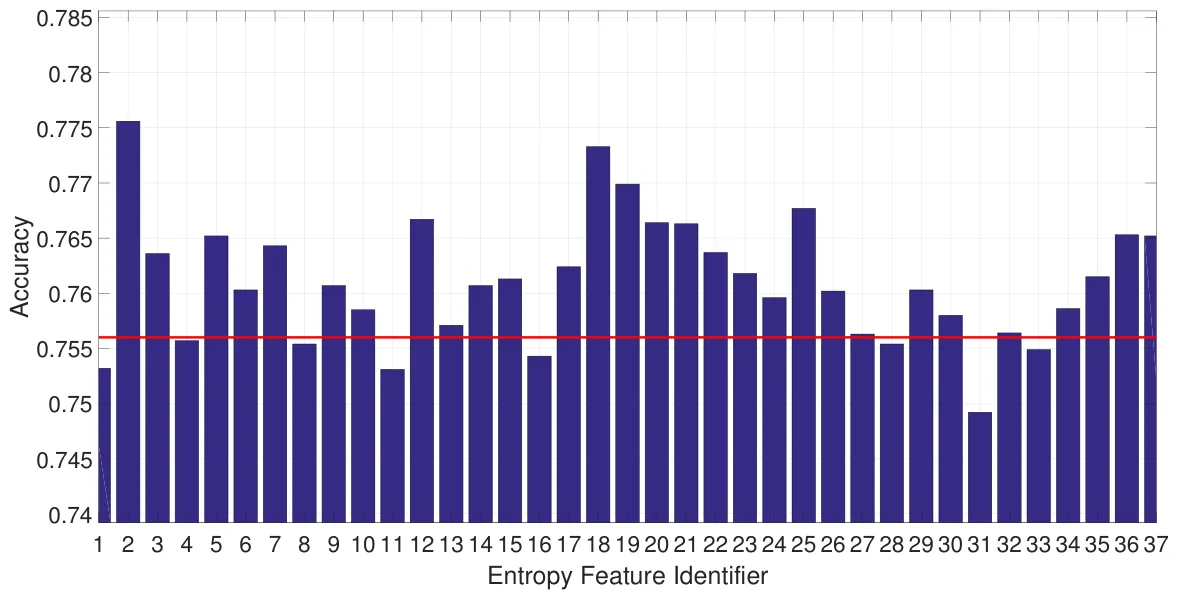
Office 1.

The main result of the study is the comparison of the use of the non-entropy features used mainly in the literature [1,5], and the use of the entropy features. More in detail, Figure 6 shows the comparison of the accuracy obtained with RF for each entropy feature combined with the non-entropy features for the first environment (Office 1). The baseline of using only the non-entropy features is the red line. These results are obtained in the $DS_{FS}$ portion of the data set.

It should be noted that the accuracy results provided are the result of the mean of 10 executions of the RF algorithm.

It can be seen in Figure 6 that most of the entropy features and in particular the bubble entropy (ID = 2) and the attention entropy (IDs 18–21) provide a significant improvement compared to the baseline.

**Figure 7:**
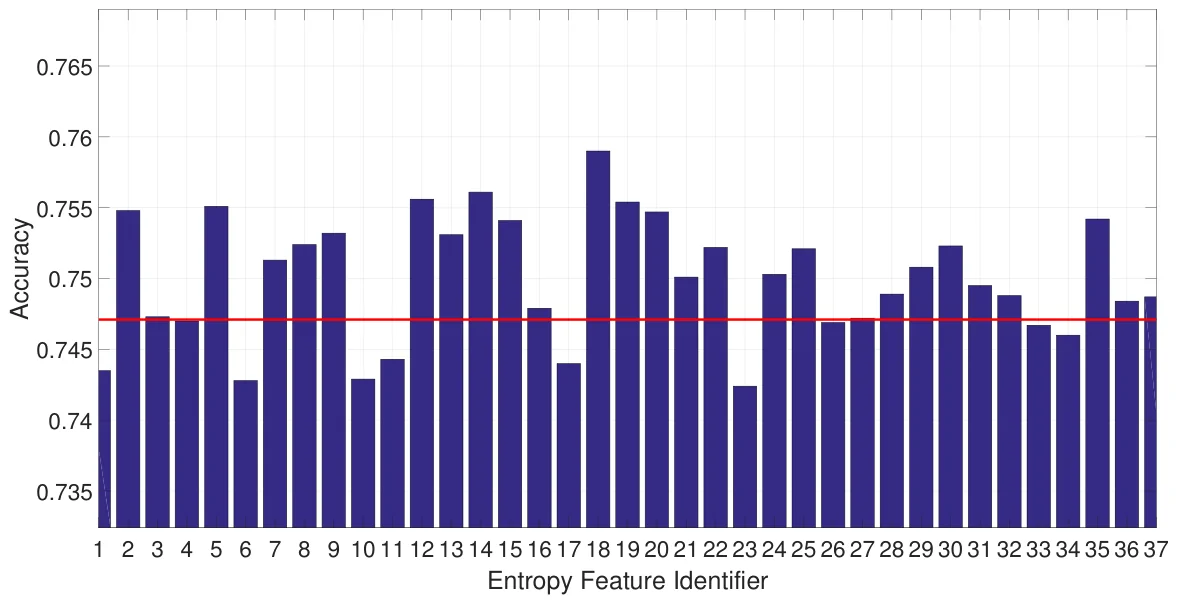
Office 2.

The remarkable improvement in the accuracy of bubble entropy and attention entropy is also visible in Office 2 in Figure 7. The propagation environment of Office 2 is supposed to be quite similar to Office 1. On the other hand, it can be seen that entropy features may have a slightly different behaviour across environments. In particular, the sample entropy (ID = 12, 14) also performs well.

Apart from the empirical results presented here, there are some potential reasons, which could justify the optimal performance of the bubble entropy and the attention entropy for the UWB LOS/NLOS classification problem. The bubble entropy is based on the bubblesort ordering algorithm. UWB impulse impacted by obstacles and reflections in their propagation path can alter the order of the values in a CIR either by attenuating the values (e.g., presence of obstacles) or by adding components at different timeframes due to reflections and general fading effects. As a consequence, the result of the bubble entropy will be different between LOS and NLOS conditions. Attention entropy is based on a completely different design than bubble entropy but it can also exploit the impact of fading on the UWB CIR. As described before, attention entropy analyses the frequency distribution of the intervals between the key observations in a time-series. The intervals between key points in the CIR are usually significantly altered by the fading effects due to reflections, which introduce delays in the propagation path of the UWB signal. In this case, attention entropy is particularly suitable to exploit such time intervals.

**Figure 8:**
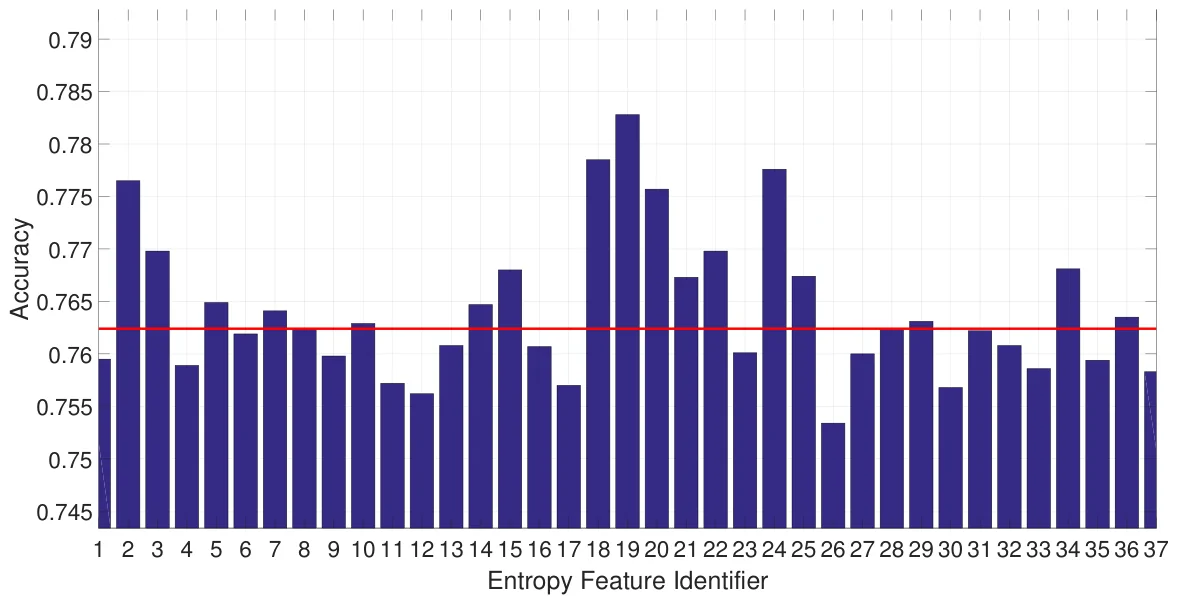
Small apartment.

Similar results are presented in Figure 8 for the small apartment propagation scenario. Again, bubble entropy and attention entropy provide a strong performance, but it can also be observed that most of the entropy features are well above the baseline of non-entropy features. In particular, the increment entropy (ID = 24) also has a performance comparable to the attention entropy.

**Figure 9:**
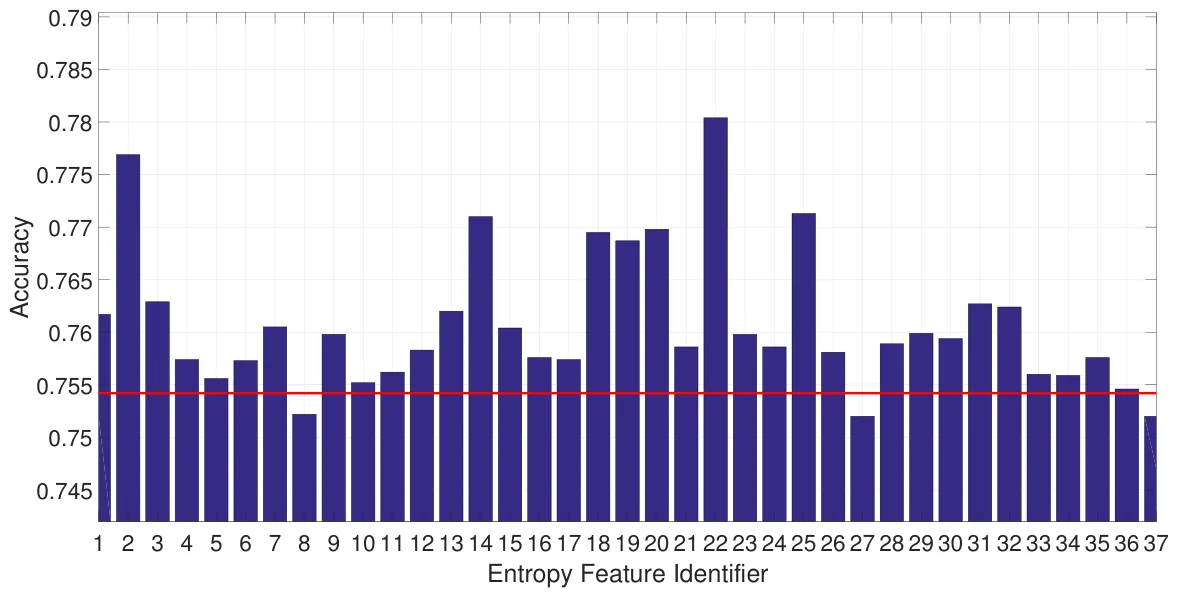
Small workshop.

**Figure 10:**
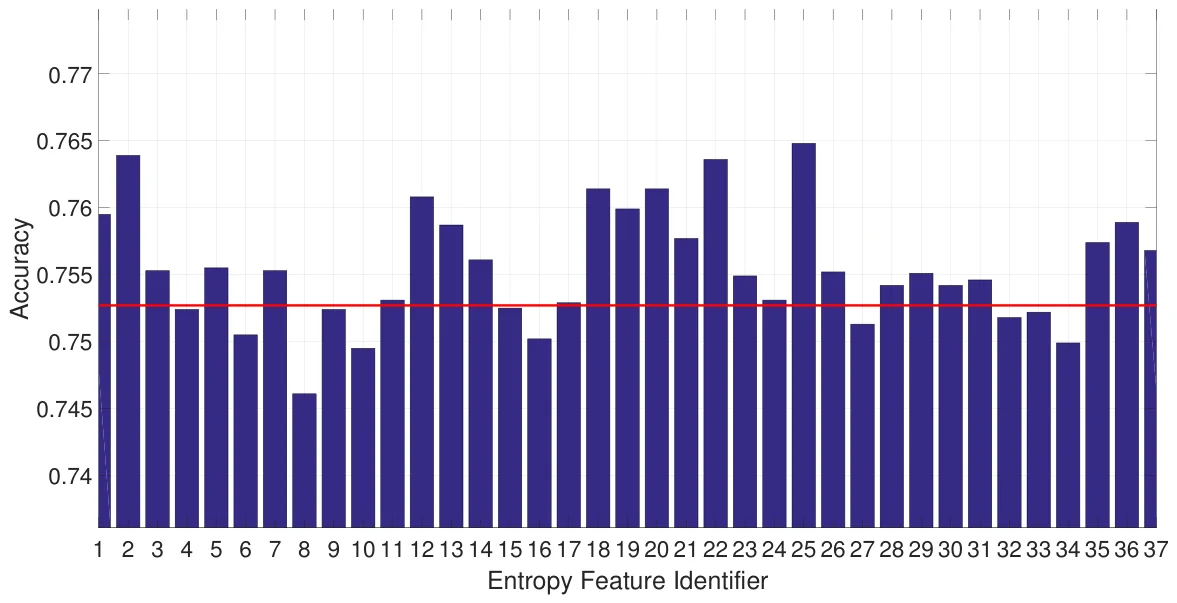
Kitchen.

**Figure 11:**
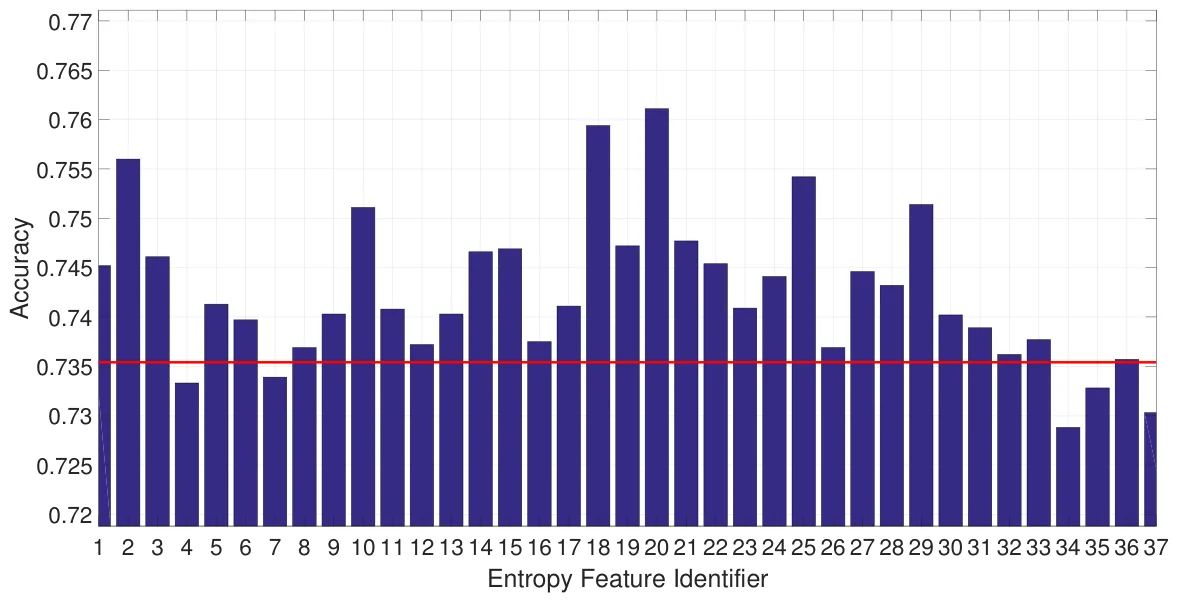
Bedroom.

**Figure 12:**
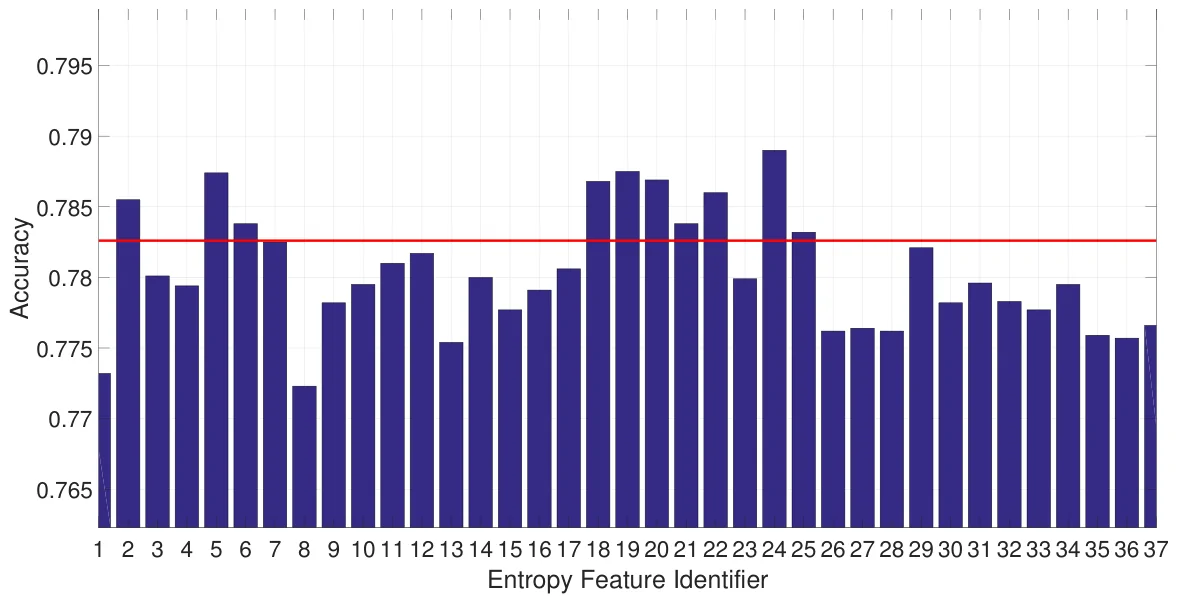
Boiler room.

Similar results are also obtained for the propagation environments of the small workshop (Figure 9), the kitchen (Figure 10) and the bedroom (Figure 11), where some other entropy measures also provide excellent performance in comparison to the bubble entropy and attention entropy mentioned above. For example, sample entropy (ID = 12, 13) in the kitchen environment. The final propagation environment is the Boiler room and is shown in Figure 12. The results shown in this figure are slightly different from the others because most of the entropy features do not perform well against the baseline apart from the bubble entropy and attention entropy already mentioned.

**Figure 13:**
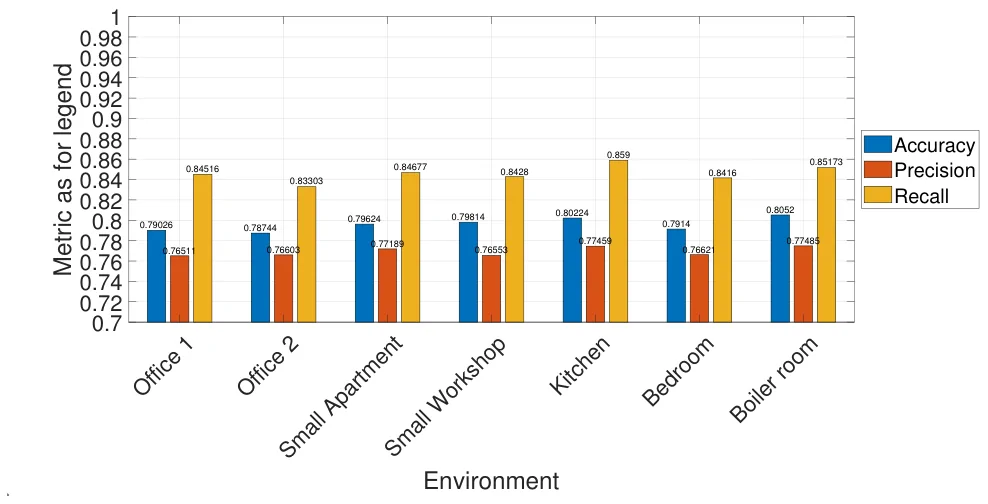
Results for accuracy, precision, and recall using the wrapper method with the five best entropy features.

In the second phase, the best entropy features identified in the previous figures are used to estimate the accuracy, precision and recall of the UWB LOS/NLOS condition. For this set of results, the portion of the data set $DS_{RS}$ (and the related feature space $FS_{RS}$) was used. As described before, the sets of 5 best, 10 best, 15 best and the entropy measures whose accuracy was higher than the baseline of the non-entropy measures are used. The accuracy, precision and recall for the set of five best entropy features in all seven propagation environments are shown in Figure 13. It can be seen that there is an unbalance between precision and recall, with the values of recall much higher than the precision. In most environments, the accuracy is almost 80%.

**Figure 14:**
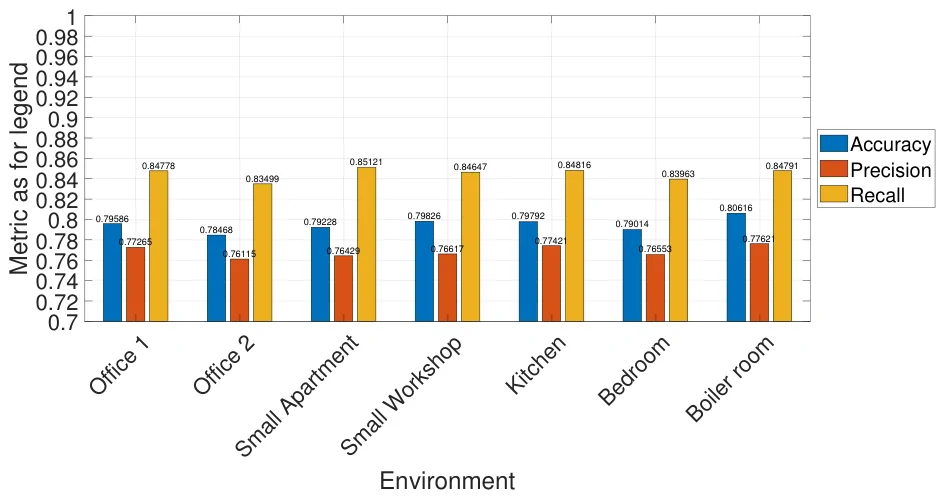
Results for accuracy, precision, and recall using the wrapper method with the 10 best entropy features.

**Figure 15:**
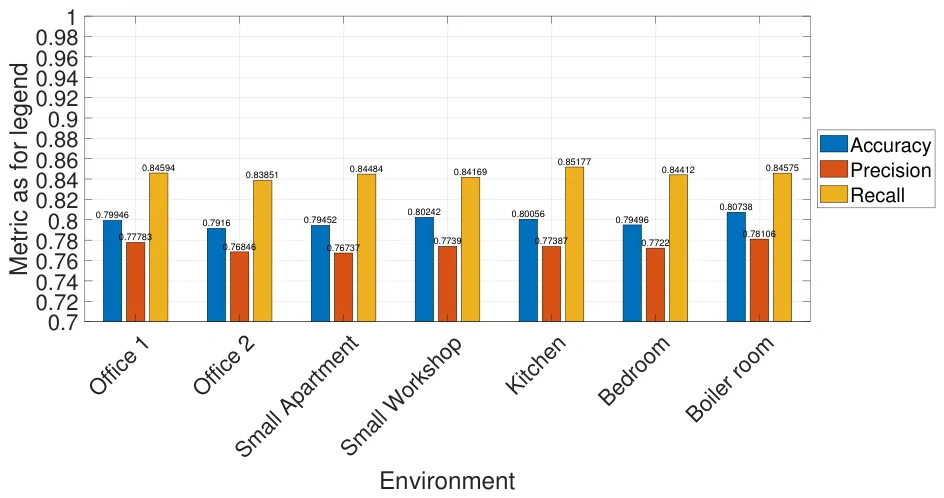
Results for accuracy, precision, and recall using the wrapper method with the 15 best entropy features.

Similar results are obtained for accuracy, precision and recall with the 10 and 15 best entropy features shown in Figure 14 and Figure 15, respectively.

**Figure 16:**
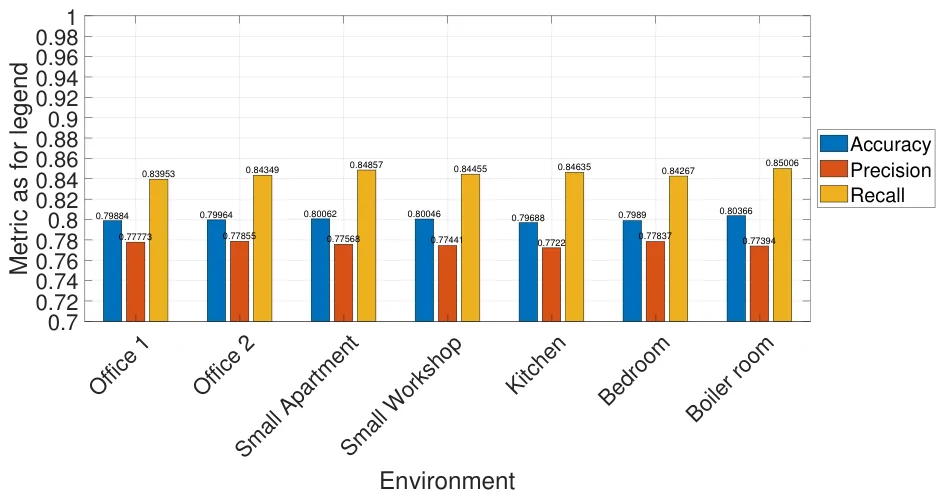
Results for accuracy, precision, and recall using the wrapper method with entropy features above the threshold of non-entropy features (i.e., BA).

The final set of results is for the set of entropy features (called BA in the rest of this paper), whose accuracy is above the non-entropy features and such results are shown in Figure 16, where it is shown that this set is able to obtain the best accuracy, precision and recall (around or above 80% for the boiler room environment) among the wrapper results considered in this section.

### 8.2. Filter Feature Selection Results

In the application of filter FSAs, the method to select the best entropy features is the cumulative weight of the highest ranking features provided by the filter FSA. Three FSAs were used: IFS, mRMR, and ReliefF.

The chosen set of entropy features are those in which the cumulative weights of the highest ranking features provided by the FSA achieve 95% of the cumulative weight of all features.

Then a preliminary analysis was performed using the FSA to identify the scores/weights provided by the algorithms themselves.

**Figure 17:**
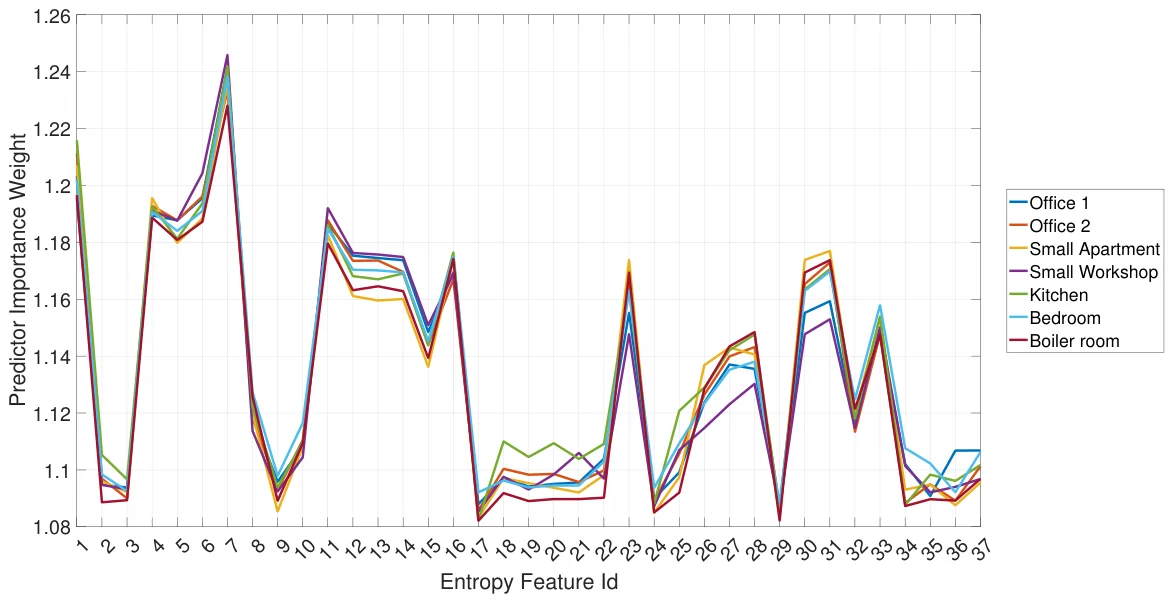
Weights estimated using the Infinite Feature Selection algorithm.

**Figure 18:**
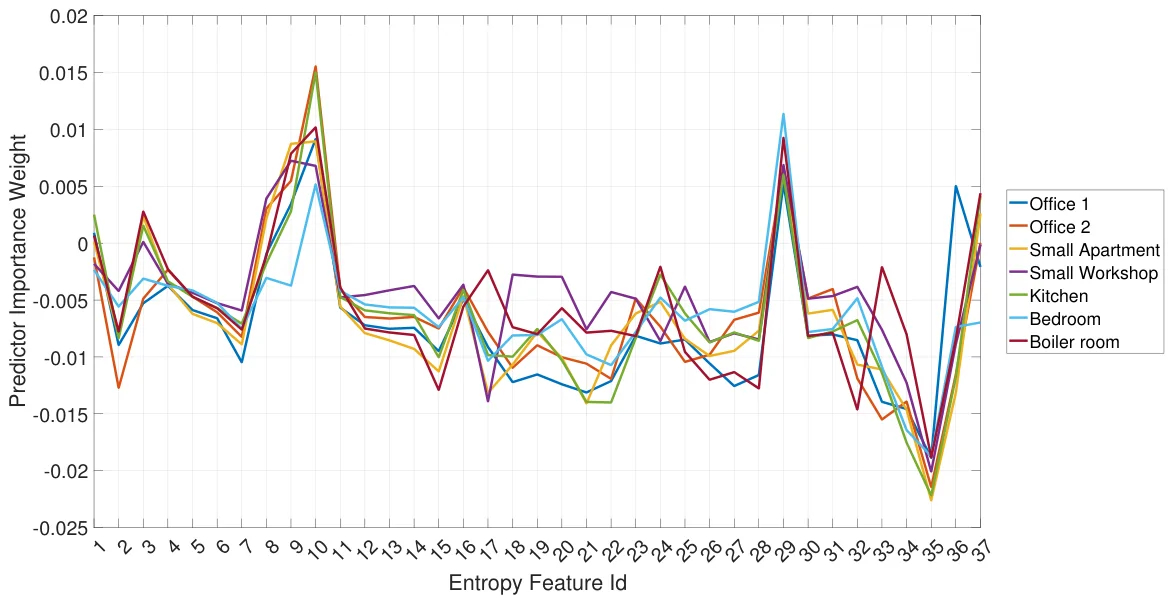
Weights estimated using the ReliefF algorithm.

**Figure 19:**
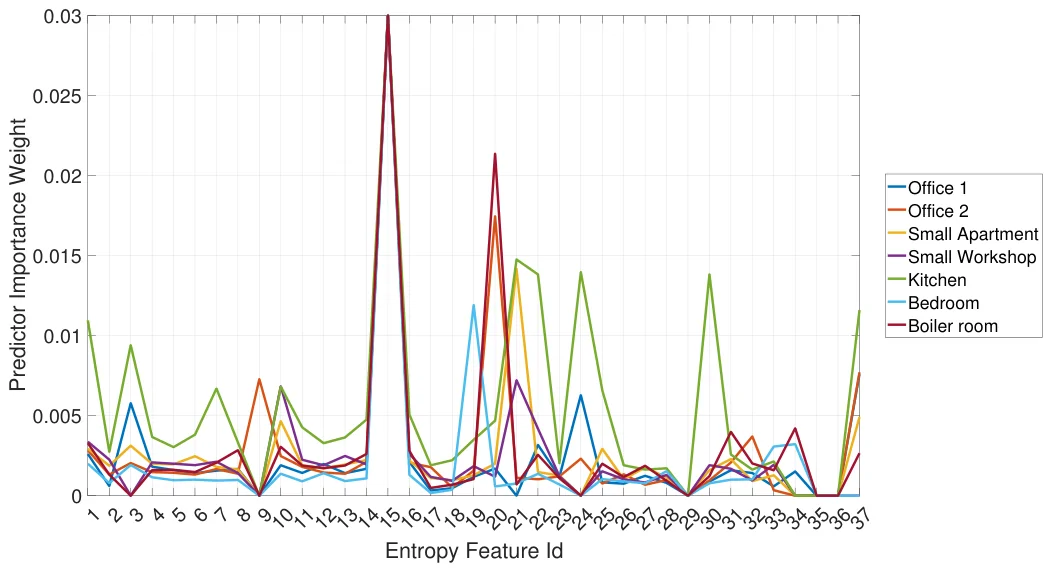
Weights estimated using the mRMR algorithm.

The results are shown in Figure 17 for the IFS algorithm, Figure 18 for the ReliefF algorithm, and Figure 19 for the rRMR algorithm. It is highlighted that the part of the data set $DS_{FS}$ was used to produce these results.

In general, the higher the predicted importance weight, the more likely the related feature is to provide a high classification accuracy. The results are somewhat different from the supervised approach presented earlier and are also not aligned among themselves. The entropy features, which are quite relevant for IFS, are not for the ReliefF algorithm or the mRMR algorithm and are also not aligned with the previous results. For example, bubble and attention entropies do not have a high predictor weight. On the other hand, for each FSA, the importance of weight/score is consistent in the different environments because the highest predictor weights are focused on specific entropy features for all environments.

Based on the results provided above, one set of optimal entropy features was selected for each FSA. It was applied to the part of the data set $DS_{RS}$ to generate the accuracy, precision, and recall values.

**Figure 20:**
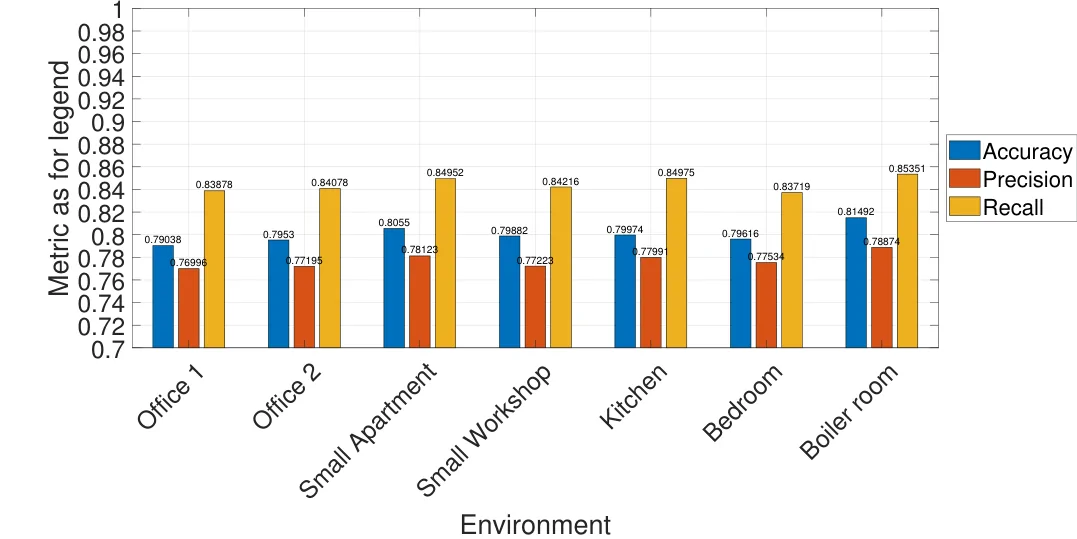
Filter FSA results for accuracy, precision, and recall using the ReliefF algorithm.

**Figure 21:**
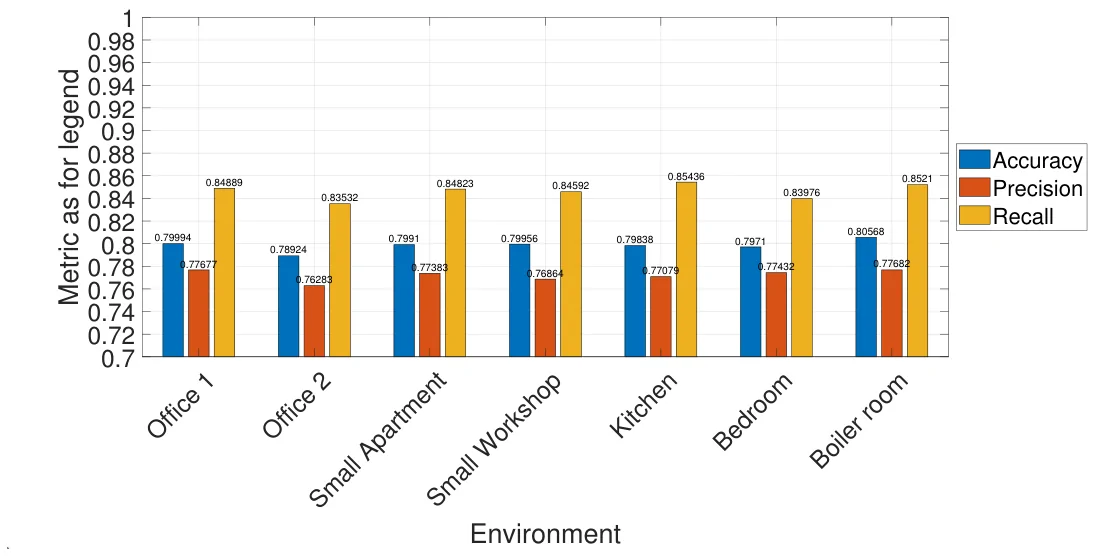
Filter FSA results for accuracy, precision, and recall using the IFS algorithm.

**Figure 22:**
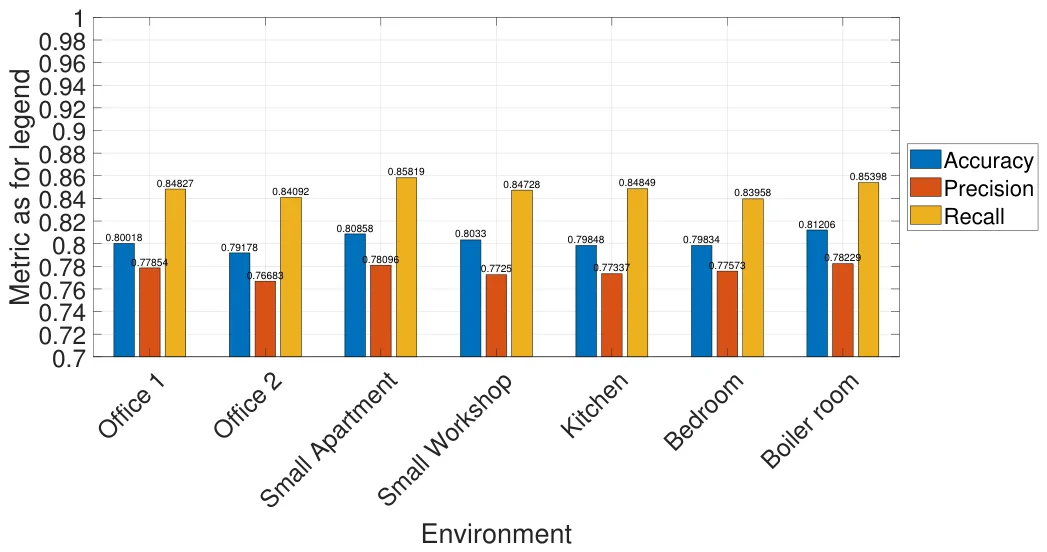
Filter FSA results for accuracy, precision, and recall using the mRMR algorithm.

In detail Figure 20 shows the metrics for the ReliefF algorithm, Figure 21 shows the metrics for the IFS algorithm, and Figure 22 shows the metrics for the mRMR algorithm. The results are consistent across the environments, with IFS obtaining the best score for the first environment (Office 1) and mRMR and ReliefF obtaining the best score for the small apartment and the boiler room environment. It is noted that in most environments, the entropy-based approach is able to reach or overcome (on the basis of the boiler room environment) 80% accuracy, which is a remarkable achievement when compared with the results in the literature for the eWine data set. A detailed comparison of these results with the results in the literature is provided at the end of this Results section in Section 8.5.

In summary, it can be seen that the entropy features provide a significant improvement for all environments. This justifies the proposal to use entropy measures for this problem.

### 8.3. Instance Selection Results: Choice of Entropy Features Using the Wrapper Feature Selection Method

This sub-section presents the results for the application of the instance selection using the wrapper method. As mentioned above, in this case, the sets of the best 5, 10 and 15 features and BA identified with the $DS_{FS}$ portion of the data set is used to implement instance selection. Note that the number of optimal entropy features is variable in BA depending on the propagation environment. The classification with the selected instance is done on the $DS_{RS}$ portion of the data set.

**Figure 23:**
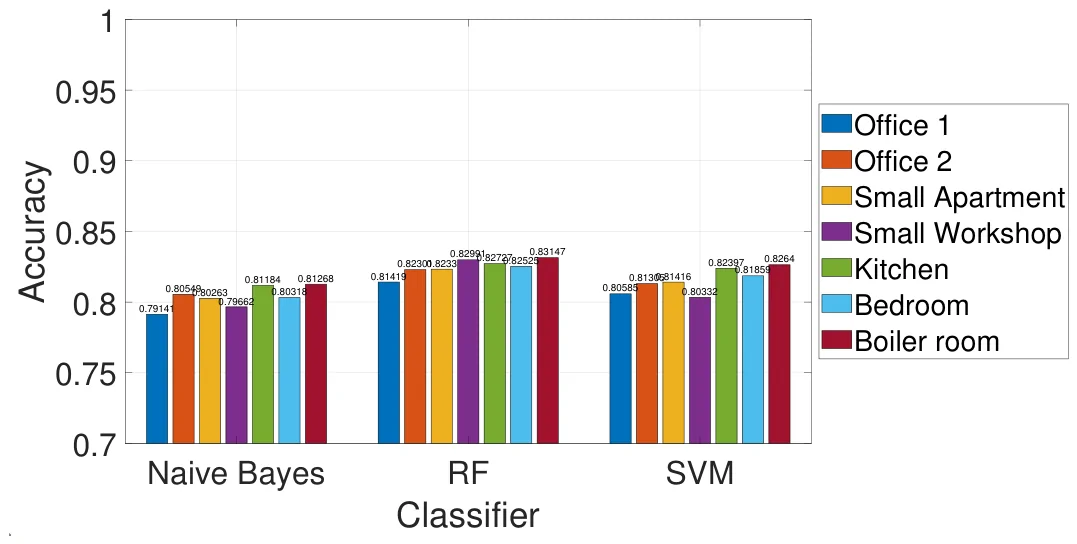
Accuracy with best five entropy features.

**Figure 24:**
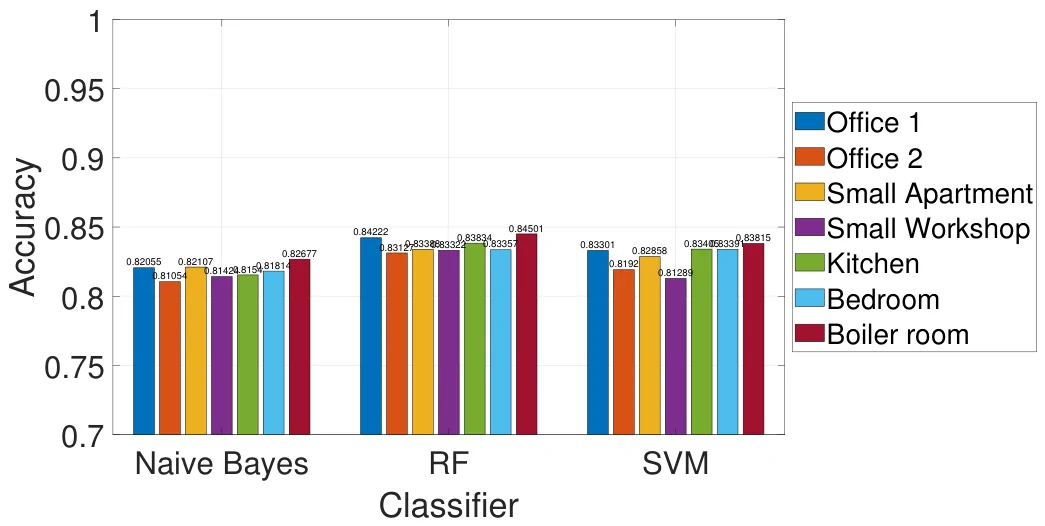
Accuracy with best 10 entropy features.

**Figure 25:**
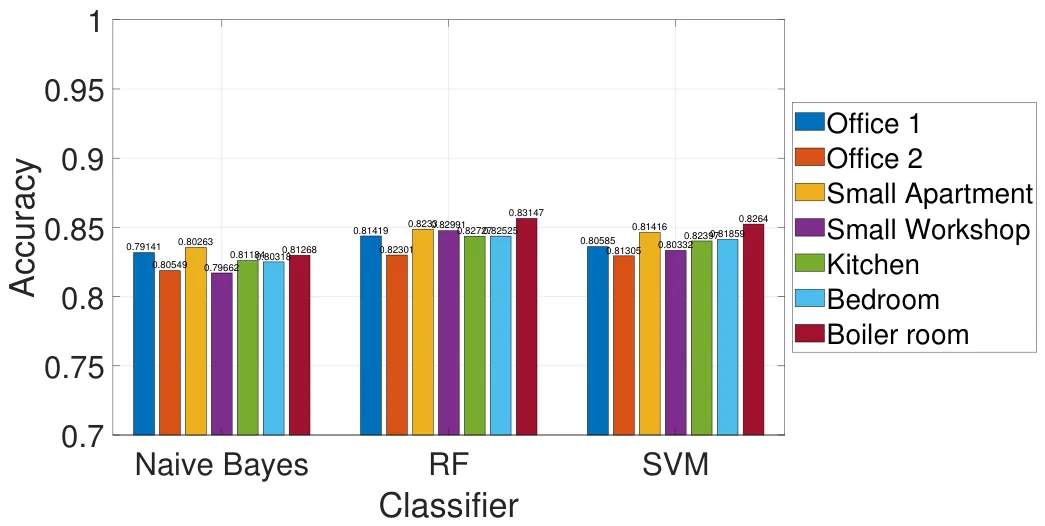
Accuracy with best 15 entropy features.

**Figure 26:**
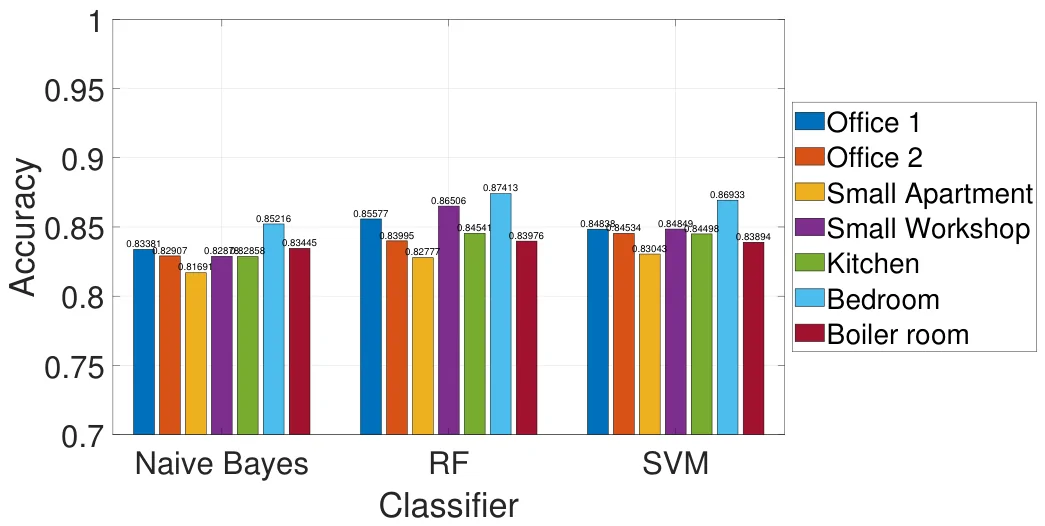
Accuracy with the BA features.

Figure 23, Figure 24, Figure 25 and Figure 26 present the accuracy with Naive Bayes, RF and SVM for the sets of best 5, 10, 15 and BA features, respectively. It can be seen that the classification accuracy is optimal using the RF classifier and that the accuracy increases with a larger set of features even if the optimal results are obtained with the BA set of entropy features. This is because the larger the set, the higher is the power of the ensemble method to remove the noisy or non-discriminative samples from the data set. The other result, which can be seen from these figures, is that precision is greatly increased (more than 5% depending on the propagation environment) thanks to the instance selection procedure compared to the results obtained using the best entropy metrics shown in the previous Section 8.1.

**Figure 27:**
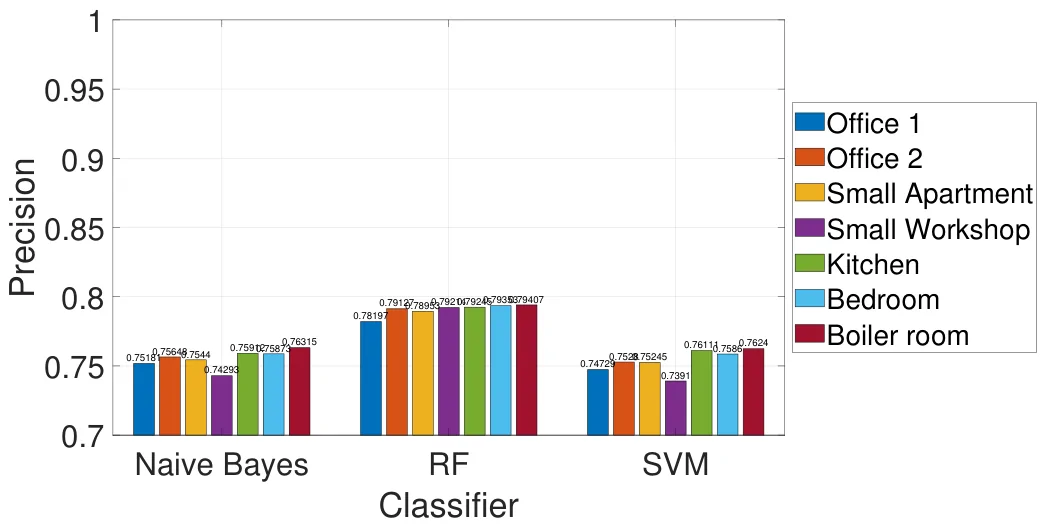
Precision with best five entropy features.

**Figure 28:**
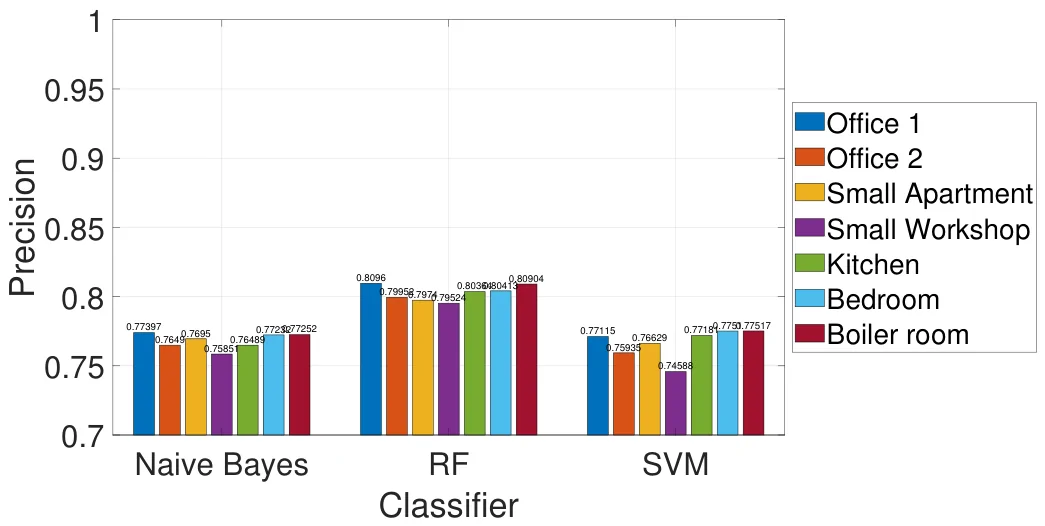
Precision with best 10 entropy features.

**Figure 29:**
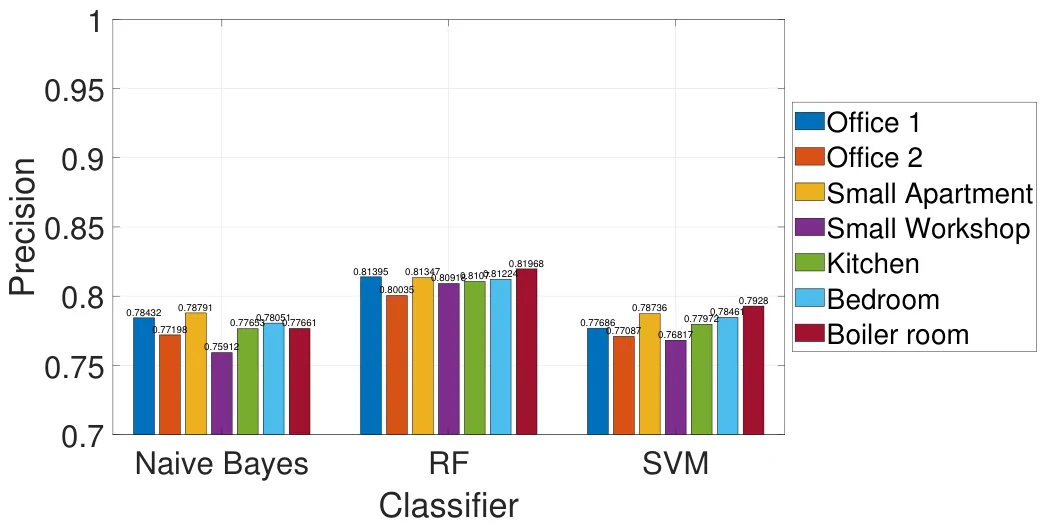
Precision with best 15 entropy features.

**Figure 30:**
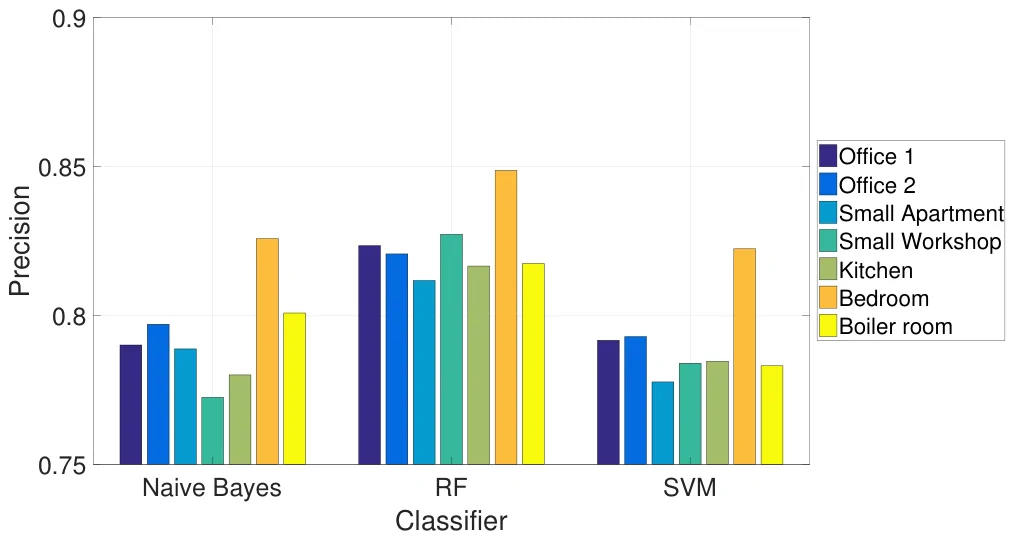
Precision with the BA features.

The following Figure 27, Figure 28, Figure 29 and Figure 30 show the precision for the sets of the best 5, 10, 15 and BA features. It can be seen that the precision is slightly lower than the accuracy, but the RF classifier still performs better compared to the Naive Bayes and SVM.

**Figure 31:**
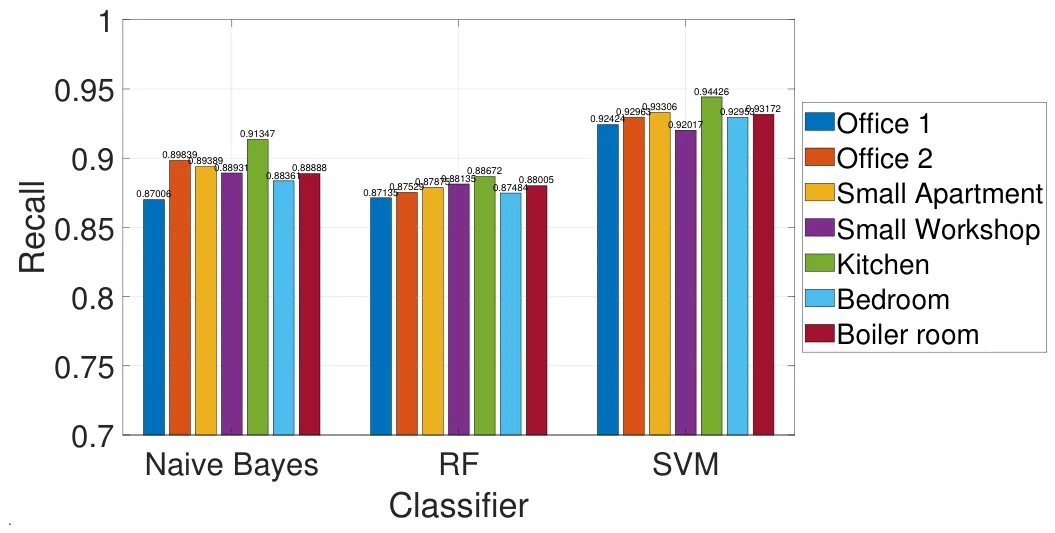
Recall with best five entropy features.

**Figure 32:**
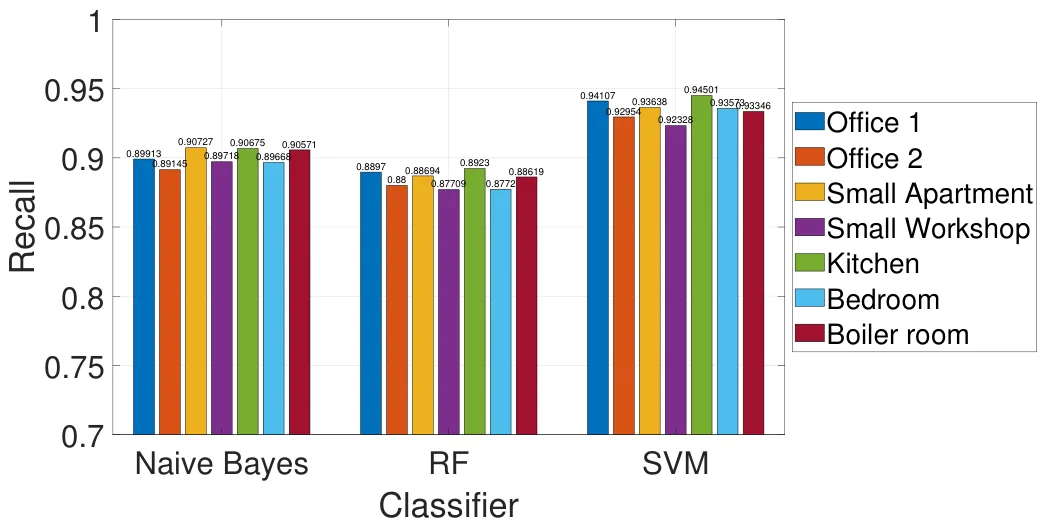
Recall with best 10 entropy features.

**Figure 33:**
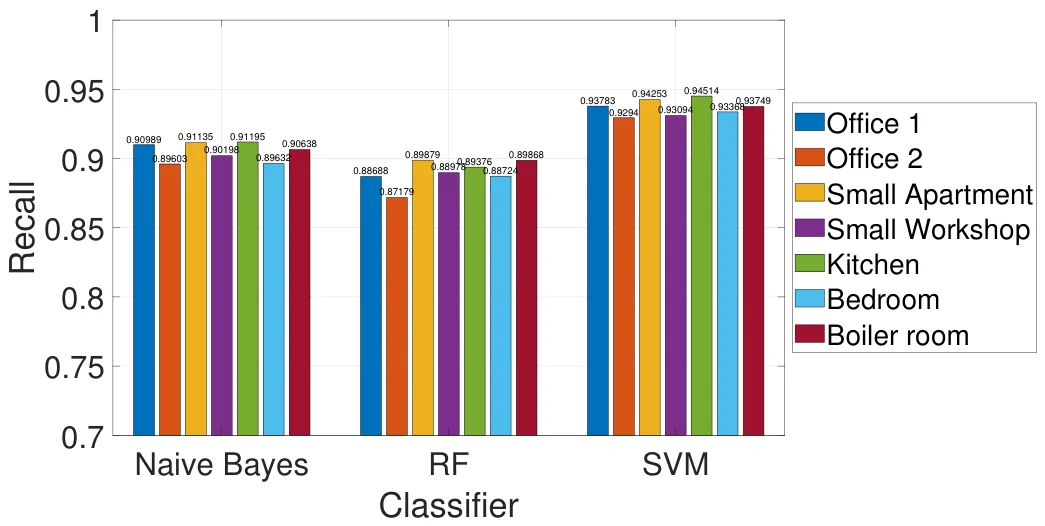
Recall with best 15 entropy features.

**Figure 34:**
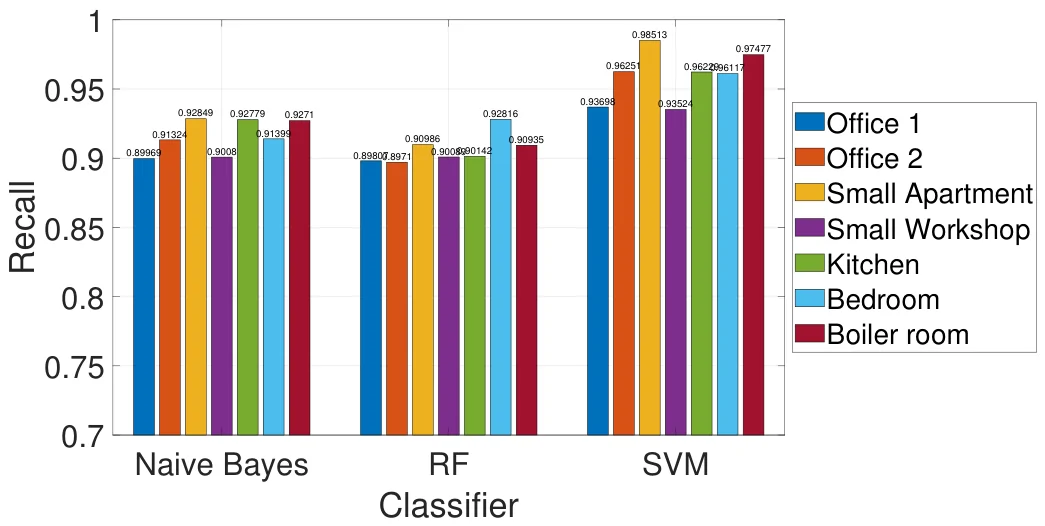
Recall with BA features.

Different results are obtained with the recall metric as shown in Figure 31, Figure 32, Figure 33 and Figure 34 for the sets of best 5, 10, 15 and BA features, respectively. The SVM and the Naive Bayes have better recall than the RF classifier. Depending on the practical application of the LOS/NLOS classification, a user may prefer to use SVM instead of the RF classifier if recall is a more important metric.

**Figure 35:**
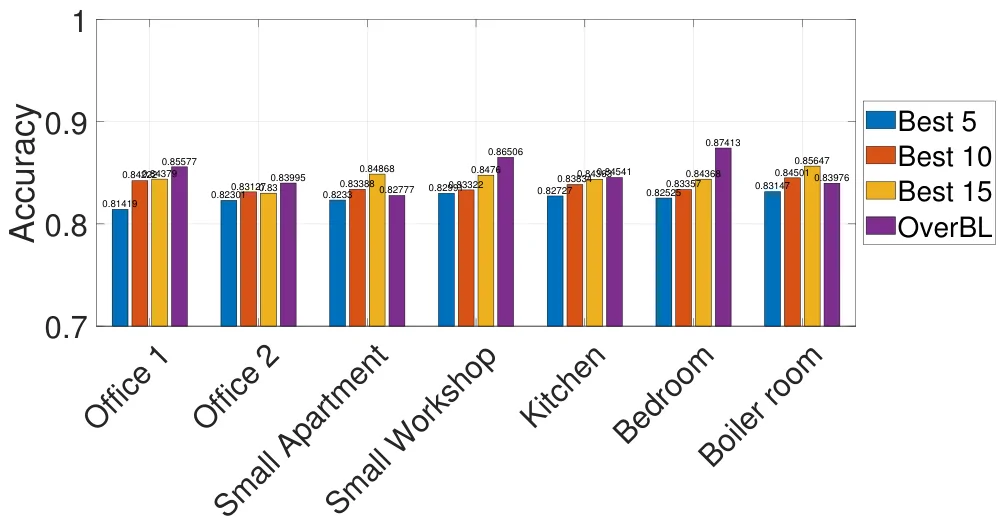
Accuracy comparison among the wrapper sets of features.

**Figure 36:**
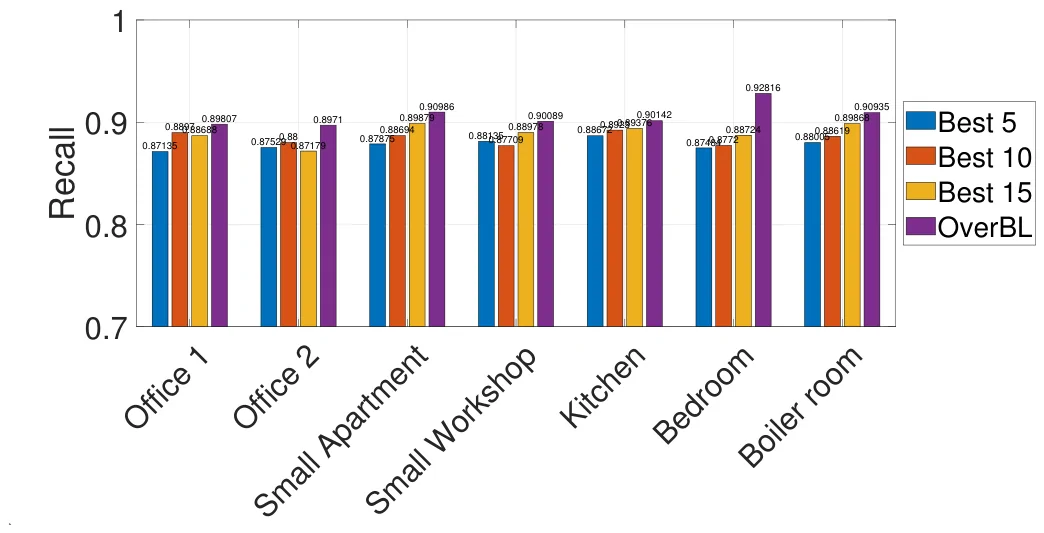
Recall comparison among the wrapper sets of features.

**Figure 37:**
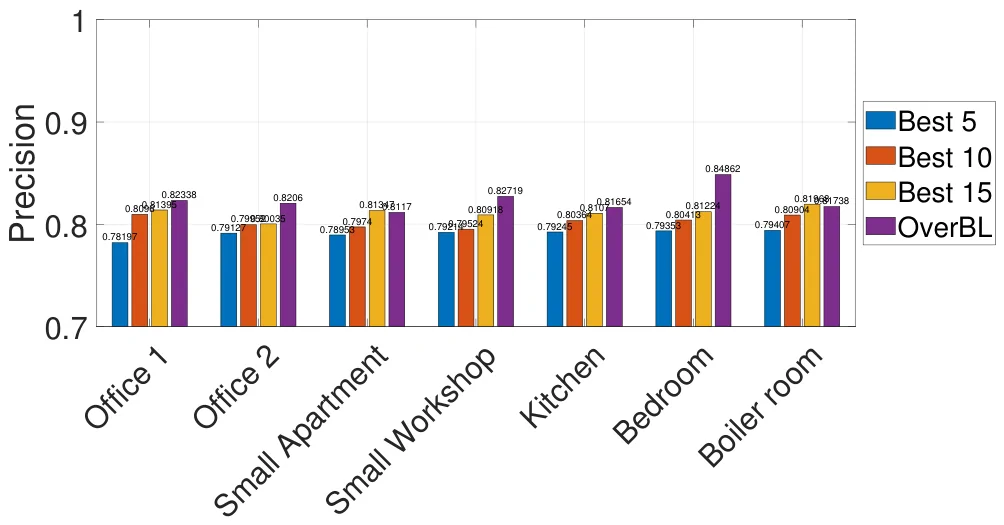
Precision comparison among the wrapper sets of features.

The trend that an increase in the number of entropy features in general produces better performance can also be seen in the following figures, which presents the same results shown in the previous figures but with the perspective of comparing the set of features using the RF classifier. In detail, Figure 35, Figure 36 and Figure 37 show the accuracy, precision, and recall in the seven different environments, respectively. Even if in general, the BA set of features has the best accuracy, in the small apartment and boiler room environments with the set of 15 best features, the accuracy is slightly higher than BA. Similar results are obtained for precision, while the BA is always better than the other set of features for the recall metric.

### 8.4. Instance Selection Results: Choice of Entropy Features Using Filter Feature Selection Method

This sub-section presents the results for the application of the instance selection algorithm using the entropy measures identified by the different FSAs with the rule that the cumulative weight/score of the highest ranking features exceed the threshold of 95% of the overall weight/score of all the features.

**Figure 38:**
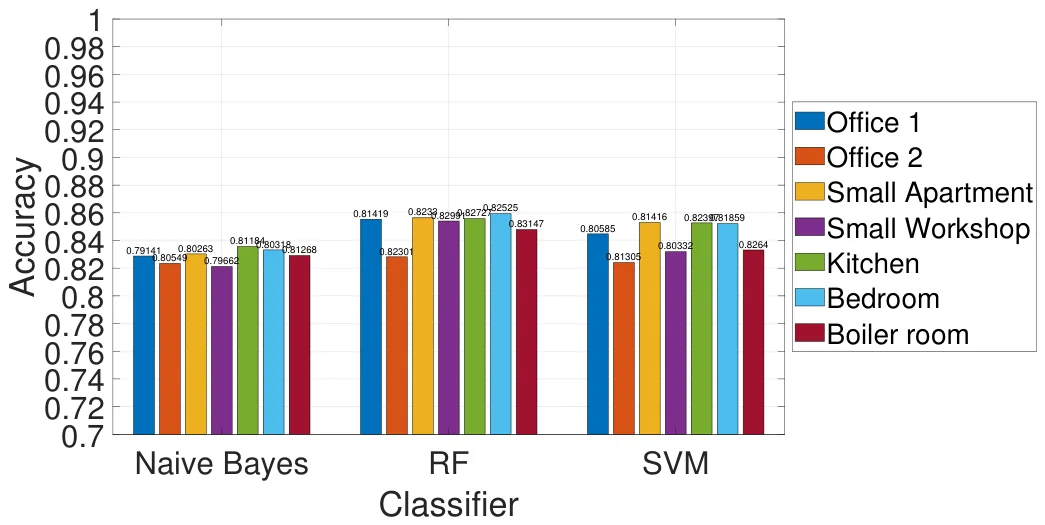
Accuracy with entropy features identified with the IFS algorithm.

**Figure 39:**
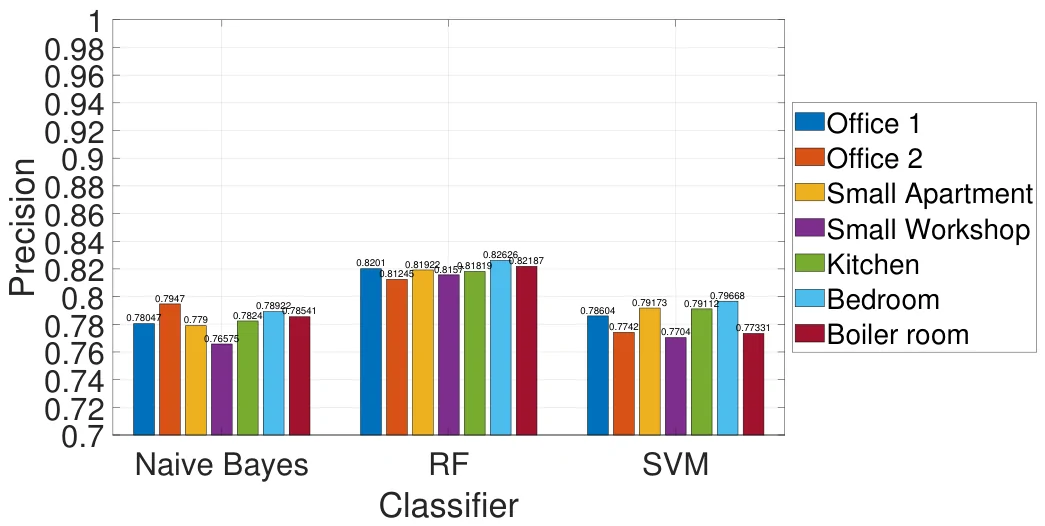
Precision with entropy features identified with the IFS algorithm.

**Figure 40:**
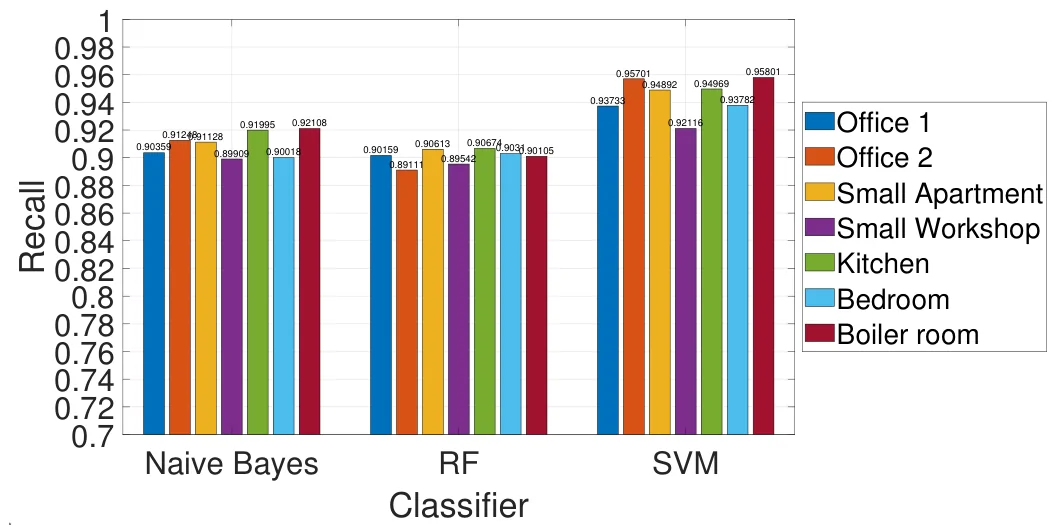
Recall with entropy features identified with the IFS algorithm.

In detail, Figure 38, Figure 39 and Figure 40 show the precision, precision, and recall values obtained using the instance selection algorithms and IFS FSA in the different propagation environments, respectively. It is noted that the RF classifier is able to obtain the best accuracy and precision, but SVM obtains better recall values. Depending on the needs of the user of the approach, one of two classifiers may be preferable. It is also noted that for IFS FSA the improvement in classification accuracy using the instance selection is limited compared to the baseline, because there is only 1% improvement in accuracy at best.

**Figure 41:**
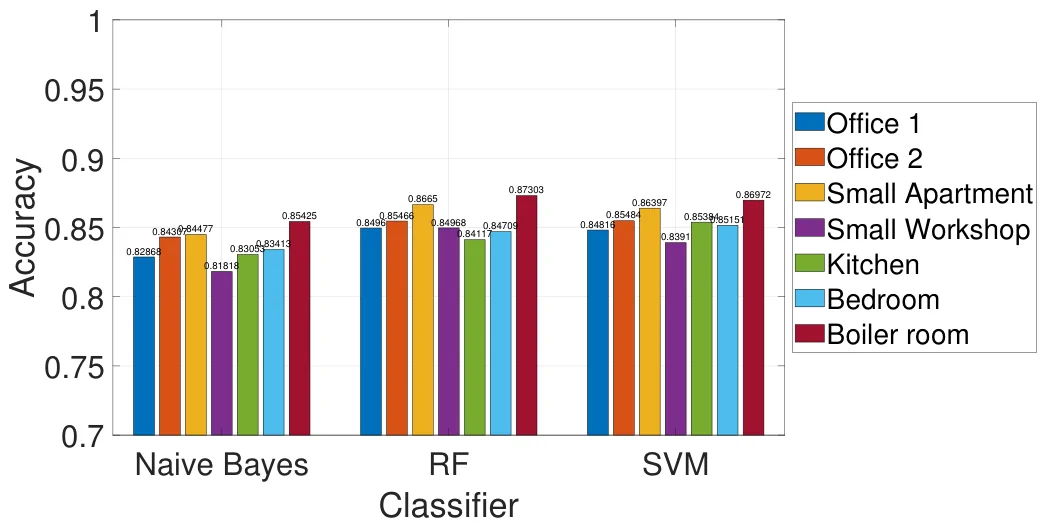
Accuracy with entropy features identified with the ReliefF algorithm.

**Figure 42:**
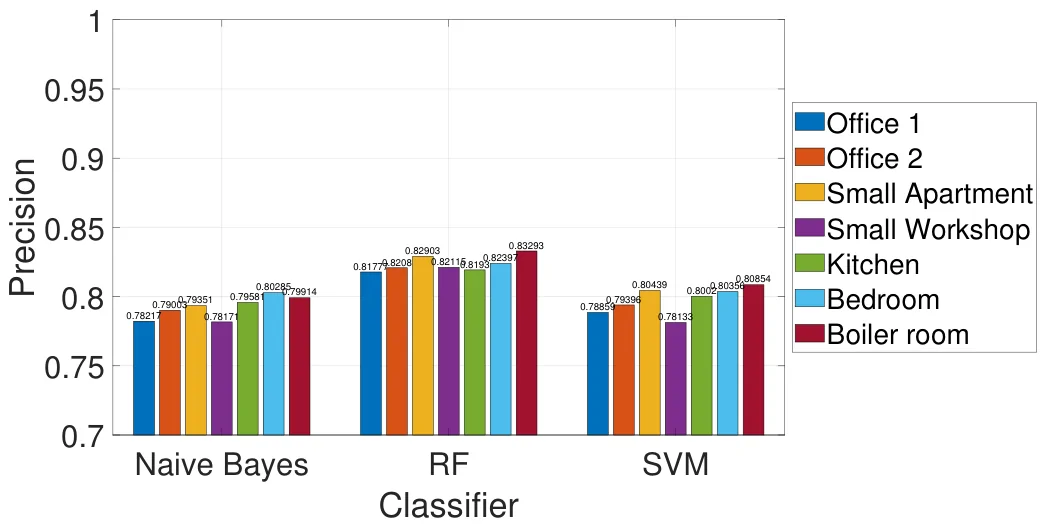
Precision with entropy features identified with the ReliefF algorithm.

**Figure 43:**
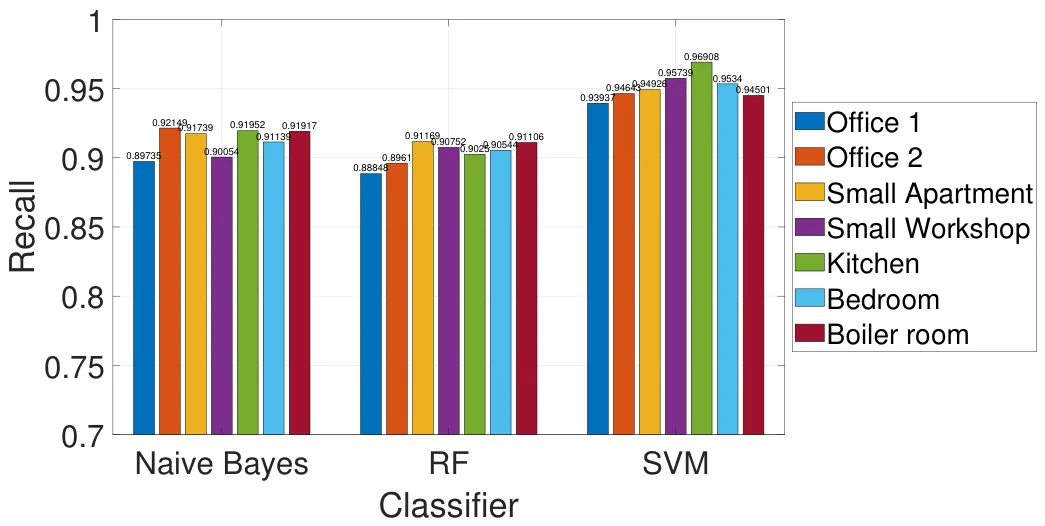
Recall with entropy features identified with the ReliefF algorithm.

Similar results are obtained using the ReliefF algorithm with the accuracy, precision and recall results presented in Figure 41, Figure 42 and Figure 43, respectively.

**Figure 44:**
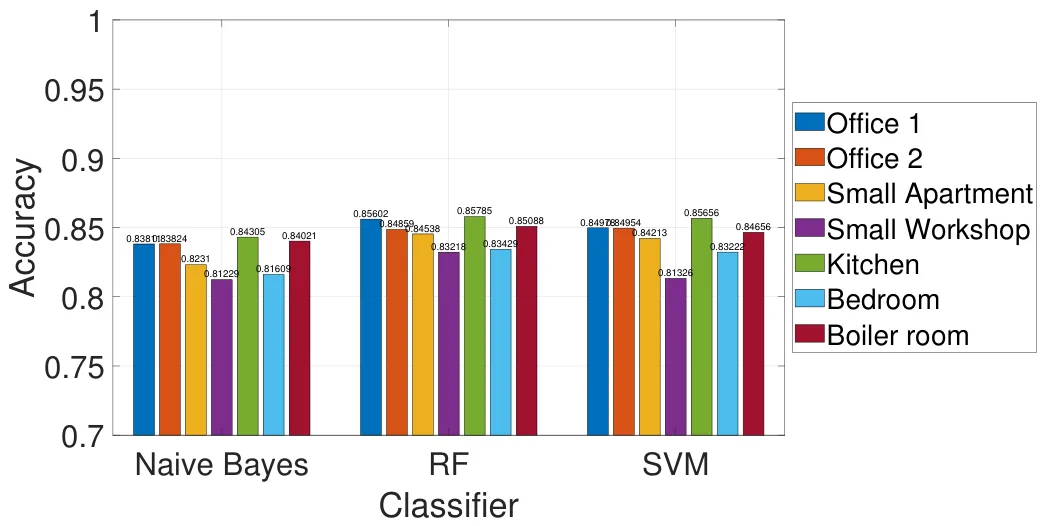
Accuracy with entropy features identified with the mRMR algorithm.

**Figure 45:**
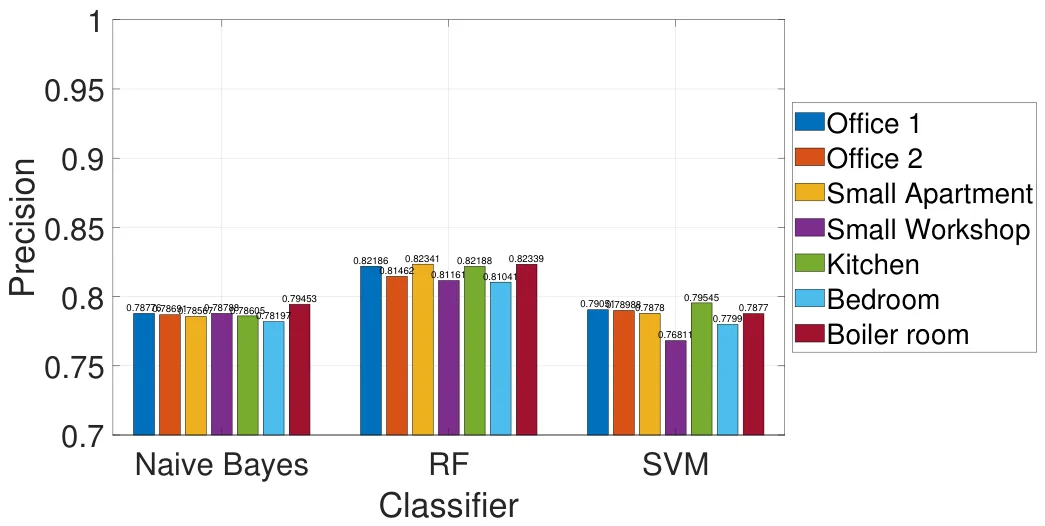
Precision with entropy features identified with the mRMR algorithm.

**Figure 46:**
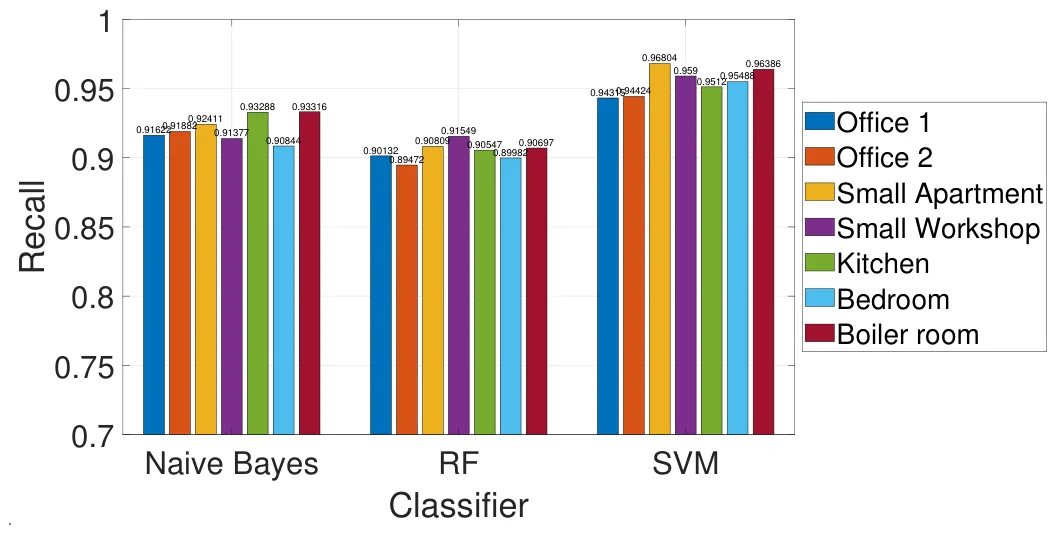
Recall with entropy features identified with the mRMR algorithm.

The best accuracy values with FSA are obtained using the mRMR algorithm shown in Figure 44, Figure 45 and Figure 46, respectively, for accuracy, precision, and recall. The performance of the RF classifier is somewhat better than SVM in accuracy and precision, but is worse for the recall metric.

As for the case of the wrapper method, the use of mRMR presents a strong improvement in accuracy using instance selection compared to the baseline. The results of the RF classifiers show that, depending on the propagation environment, the accuracy is between 83% and 86% with an improvement between 2% and 4%.

### 8.5. Summary of the Numerical Results and Comparison with the Literature on the eWine Data Set

This section provides a summary of the graphical results presented in the previous sub-sections in a numeric format. More importantly, it provides a comparison of the results obtained with the approach proposed in this paper with the results presented by similar studies in the literature on the eWine data set.

Disclaimer: the author would like to highlight that such a comparison is only for informative purposes and difference in accuracy may also depend on the algorithmic steps implemented by the specific authors in the identified papers. In particular, this study uses an ensemble instance selection approach in which the retained observations are, by design, a subset of relatively easy-to-classify samples, whereas the discarded observations are likely to include the more difficult cases in which the models are more prone to error. Therefore, improved performance in the reduced data set may partly reflect the removal of difficult or noisy samples rather than a genuine improvement in classification capability (e.g., due to sophisticated ML or DL architectures).

The results are presented in Table 5, which shows that both filter and wrapper FSAs provide excellent results reaching a maximum classification accuracy of 85.44% for the filter FSAs with the IFS and the SVM classifier and 84.97% with the BA and the RF classifier. Such results not only largely outperform the ML and Supervised Time Series Forest applications on the same eWine data set, but also the application of some DL architectures like CNN, LSTM, CNN+Bi-LSTM and CNN plus denoising. Only very recent DL architectures like ADCNN-BiLSTM and ADCNN-BiLSTM with transformer in addition to the recent LighMamba DL outperform the results presented in this study based on entropy measures. Comparison with the literature shows that entropy measures have significant potential in the application of UWB LOS/NLOS classification.

**Figure 47:**
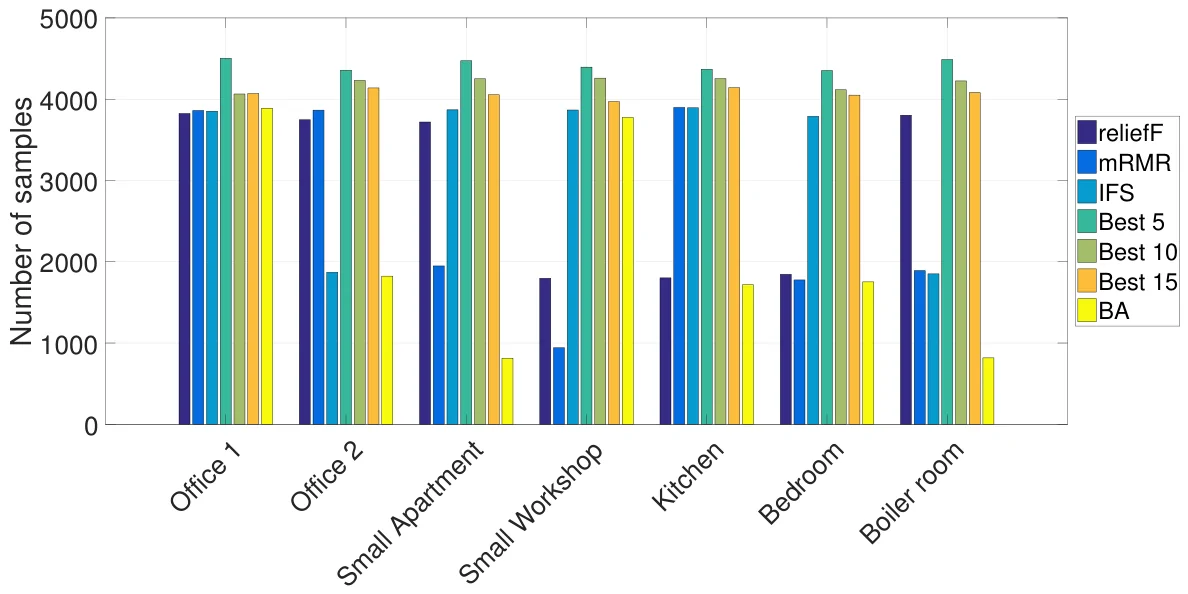
Number of samples selected by the specific UWB LOS/NLOS propagation environments and the specific instance selection FSA approach.

To complement the previous results, Figure 47 shows the number of samples identified by the instance selection algorithm for all seven environments and the different FSAs used in this study. It can be seen that the proposed instance selection algorithm is able to maintain a balance in most cases between the need to filter out the noisy and non-discriminatory samples and preserve a good number of samples. It is noted that there is high variability in the number of samples between the propagation environments. In the case of Office 1, the selected number of instances is similar in the FSAs, while in the boiler room there is a strong unbalance. This may be due to the consideration that the boiler room contains more noisy samples than the Office 1 environment.

### 8.6. Hyper-Parameter Analysis

This section provides insights on the impact of some of the hyper-parameters on the classification performance. In particular, it is evaluated the impact of the embedding factor *m* and the random forest classifier parameters. An exhaustive analysis on the impact of all the hyper-parameters for all the propagation environments and all the entropy metrics is not possible for reasons of space. The author has then selected two entropy metrics: bubble entropy and sample entropy for the analysis of the impact of the embedding parameter *m*.

**Figure 48:**
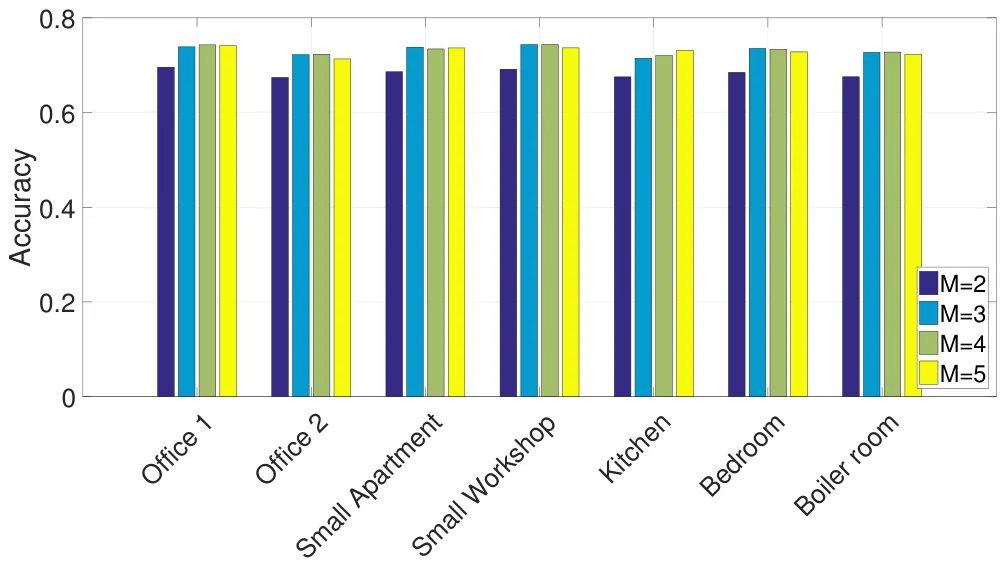
Bubble entropy.

**Figure 49:**
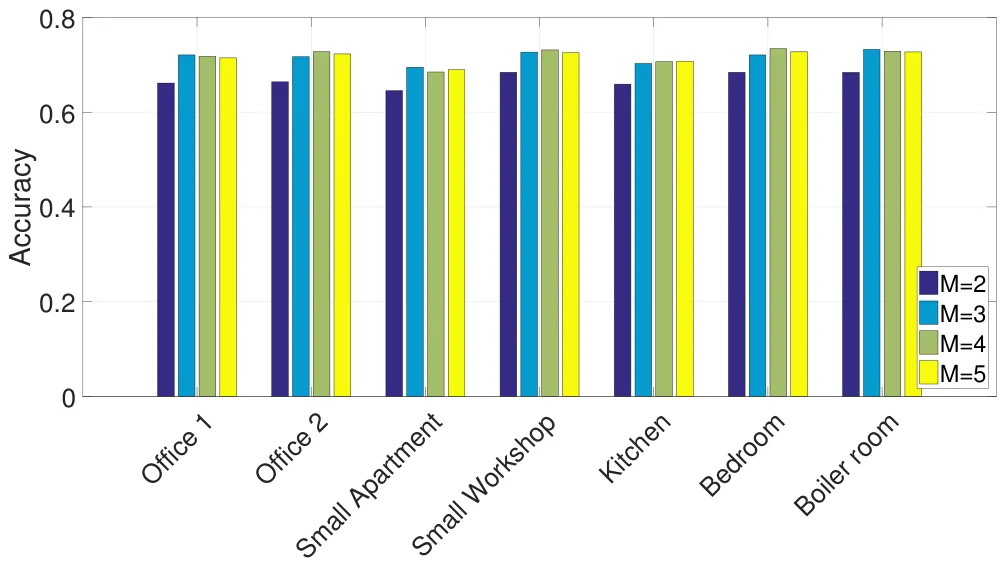
Sample entropy.

The analysis is shown in Figure 48 and Figure 49, respectively, for the bubble and sample entropy for values of *m* = 2, 3, 4, 5. It can be seen that a relative lower accuracy is obtained with m = 2 in all environments for both entropy measures, while differences in the values of *m* = 3, 4, 5 are minimal. Considering that an increase in the value of the parameter m increases the computing complexity, a value of *m* = 3 or *m* = 4 was chosen for most of the entropy measures. These results were obtained on the $DS_{FS}$ portion of the data set.

The other analysis presented in this subsection is related to the hyper-parameters classifiers. In particular, the impact of the ensemble algorithm (not to be confused with the ensemble algorithm for example selection) and the number of maximum splits is shown in the Random Forest classifier and the parameter C and kernel scale of the Radial Basis Function (RBF) in the SVM classifier. These results were obtained on the $DS_{RS}$ portion of the data set.

**Figure 50:**
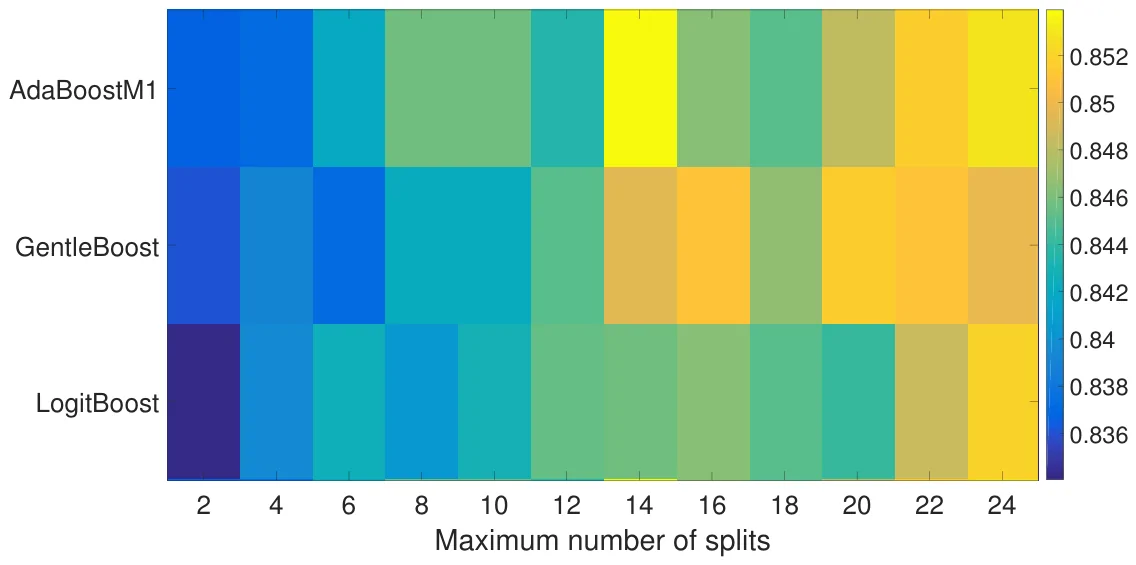
Optimisation of the random forest.

An exhaustive analysis of the impact of such classifier parameters for all filter and wrapper FSAs and for all propagation environments is not possible for space reasons. Figure 50 presents the results for the optimisation of the Random Forest ensemble classifiers with the wrapper selection of the feature ‘Best above the baseline’ and the last propagation environment ‘the boiler room’. It can be seen that the optimal algorithm is the AdaBoost M1 and a maximum number of splits equal to 14. Similar results were obtained for the other combination of features. To harmonise the computing configuration, the final parameters of the Random Forest were set to AdaBoost M1 and the maximum number of splits equal to 12 in all configurations.

**Figure 51:**
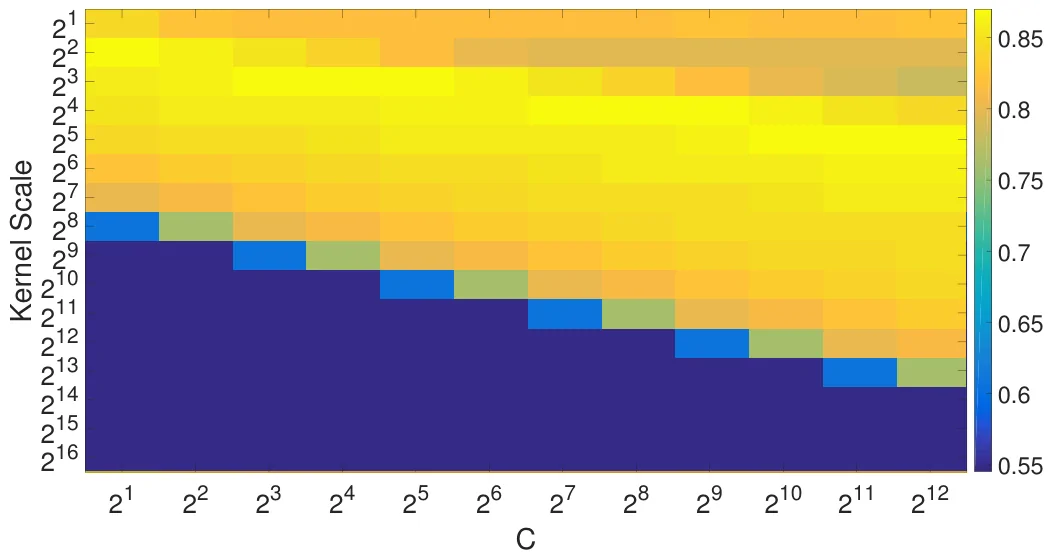
Optimisation of SVM.

In a similar way and for the same configuration of the wrapper selection of the feature ‘Best above the baseline’ and the last propagation environment ‘the boiler room’, Figure 51 shows the optimisation of the box constraint parameter C and the kernel scale of the RBF in the SVM classifier. The results show that the best accuracy values are in an area above $C=2^{6}$ and on a kernel scale below $2^{8}$. To harmonise the computing configuration, the final parameters of the SVM were set to $C=2^{8}$ and the kernel scale of the RBF to $2^{6}$ in all configurations.

### 8.7. Robustness Analysis

This section reports the results on the evaluation of the robustness of the proposed approach in presence of noise. To evaluate how much the classification performance is degraded in presence of noise, AWGN was applied to the initial data set with different values of SNR in dB: −10 dB, 0 and 10 dB. The *awgn* function of the MATLAB signal processing toolbox was used with the measured option where the signal noise level (i.e., the CIR) is measured before the noise is applied to reach the desired SNR in dB (e.g., 0 dB).

**Figure 52:**
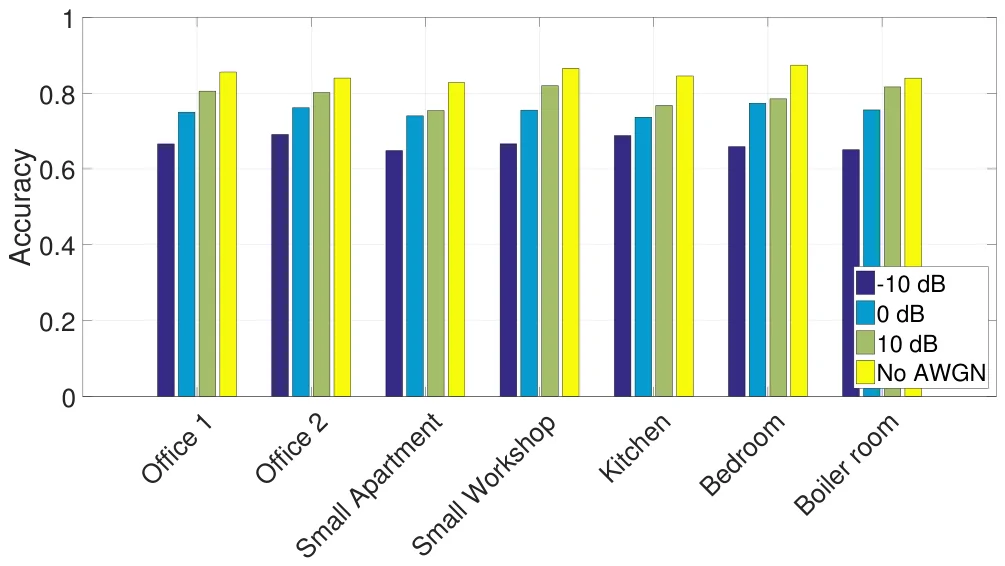
Degradation of accuracy with the wrapper FSA method (best features above the baseline).

**Figure 53:**
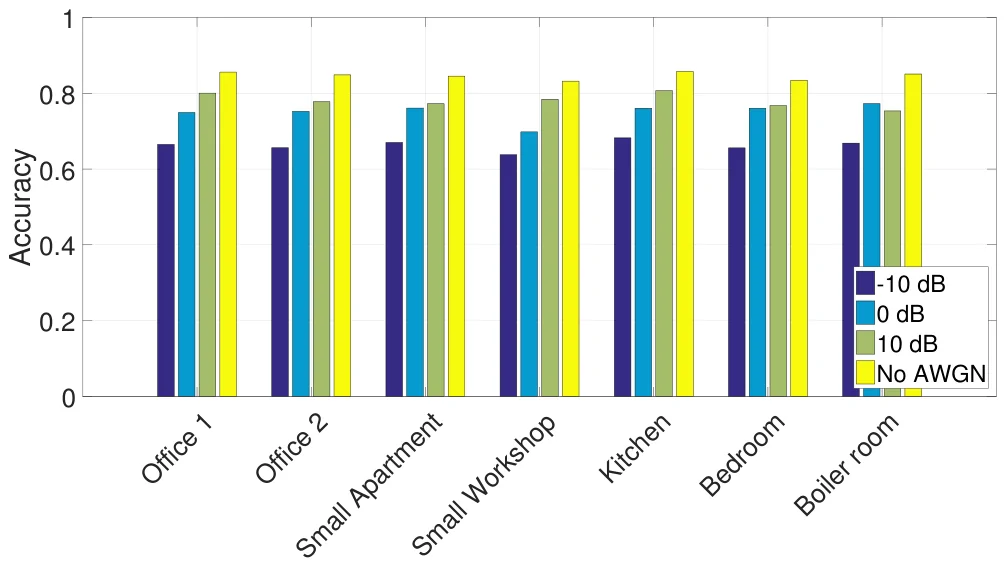
Degradation of accuracy with the filter mRmR FSA method.

The results for the accuracy are presented in Figure 52 and Figure 53, respectively for the set of features identified with the wrapper method (Best above) and the filter FSA mRmR. Similar results were generated for the other filter and wrapper selections, but they are not shown here for reasons of space.

The results show that, as expected, the application of AWGN gradually decreases the classification accuracy of the approach to lower the values of SNR in dB. However, there is no significant difference in the gradual degradation of performance accuracy between the filter and wrapper methods. Both Figure 52 and Figure 53 also show that the degradation is similar in the propagation environments and is still above 0.6 even at SNR = −10 dB.

### 8.8. Computation Time Analysis

Finally, we provide an estimate of the computing times needed to perform the following operations: calculation of the entropy and feature values, training and inference with the ML algorithms using the computing platform described in Section 4. The computing time results are presented in Table 6. The classification times are the average of the instance selection executions on the $DS_{RS}$ portion of the data set across the wrapper (BA only) and the filter executions (IFS, mRMR and ReliefF).

The application of the entropy features on 100 samples of UWB signals is approximately 8 s on the computing platform used in this study, which is a simple commercial-grade computing platform. The computing time of the entropy features is obviously much larger than the estimate of non-entropy features, but this is compensated by the much higher classification accuracy. However, the estimate of the entropy features is comparable to the training time of the classifiers used in the study. In particular, the Random Forest classifiers. The execution time of the filter FSAs (ReliefF, mRmR and IFS) on the $FS_{F}S$ portion of the data set is relatively limited in comparison to the other execution times. The memory consumption can be easily derived from the data set size (6000 samples for each of the seven propagation environments). As in other feature extraction approaches, the memory consumption of the proposed approach is one of its benefits as the initial time series of truncated CIRs of size 512 is reduced to a feature space with 54 features (a reduction of almost a factor of 10).

## 9. Conclusions and Future Developments

### 9.1. Summary

This paper has presented the results for the evaluation of entropy measures for the problem of UWB LOS/NLOS classification on the eWine public data set using various feature selection algorithms and classifiers. The results show that the application of entropy measures recently proposed in the research literature in other domains (e.g., physiological signals or mechanical processing) possess a high discriminating power in wireless communication systems as well. Such entropy measures used in an ensemble method significantly outperform approaches based on hand-crafted features or DL architecture like CNN and CNN+LSTM. More sophisticated DL architectures recently proposed in the literature still outperform our proposed approach, but with the disadvantage of significant computing complexity.

### 9.2. Limitations

The limitations of this study are identified as follows. (1) It is not known a priori which entropy measures have a higher discriminating power compared to others. At this moment, the study relies on an empirical selection method based on filter and wrapper FSAs, which may be time consuming if a large number of entropy measures are applied. On the other hand, a common trend among the eWine propagation environments is that bubble entropy and attention entropy show a significant discriminating power across environments. (2) the ensemble method is relatively simple as it is based on a unanimous voting scheme. More sophisticated weighted ensemble algorithms may provide higher performance, but this is left to future developments. (3) As with other instance selection algorithms, there is the risk of reducing the sample coverage of the UWB LOS/NLOS classification because only a portion of the overall number of samples in used. However, the instance selection algorithm also introduces the benefit of reducing the computational time of the classifier, which is usually proportional to the number of samples in which training and inference are performed.

### 9.3. Future Developments

There are various potential evolutions of this study. As mentioned before, more sophisticated ensemble algorithms (e.g., weighted voting) could be applied both among the hard labels generated by the entropy measure results or using the label probabilities returned by the classifiers. Future developments of this work may also investigate hybrid architectures, which combine entropy measures together with recently proposed DL architectures to improve the classification performance even more while trying to achieve computing efficiency. Another future development could be to expand the analysis to other entropy measures proposed in the literature beyond the ones proposed in this study. 

## Figures and Tables

**Table 1:** Description of the entropy features used in this study together with the input parameters. Part 1/3.

Id	Description	Input Parameters	Reference
1	Shannon entropy (ShEn) is the classical definition of entropy in the digital domain. It quantifies the average uncertainty or information content in a data source, measuring how unpredictable it is, with higher entropy meaning more surprise and more bits needed to encode the information. It is used as a baseline in this study	None	[37]
2,3	Bubble entropy (BubEn) is based on permutation entropy, where the vectors in the embedding space are ranked. The measure is the result of the application of the bubble sort algorithm for the ordering procedure on the input time series and the measure is the count instead of the number of swaps performed for each vector.	m = 3, τ=1	[35]
4,5,6,7	Approximate entropy (ApEn) is a measure that quantifies the regularity and unpredictability in time-series data, indicating how likely similar patterns of observations built in the embedding space of degree m are to be followed by other similar patterns. A lower value of the AE suggests more regularity (predictability), while a higher value indicates more randomness and complexity in the signal	m = 3, τ=1	[38]
8,9,10	Fuzzy entropy (FuzEn) is the negative natural logarithm of the conditional probability that two vectors similar for m points remain similar for the next m + 1 points.	m = 3, τ=1, Sigmoid function with r = [0.2, 2]	[39]
11,12, 13,14	Sample entropy (SpEn) is an evolution of ApEn, which addresses two main weaknesses of the latter entropy definition: ApEn is heavily dependent on the record length and it lacks relative consistency	m = 3, τ=1	[40]
15,16	Renyi entropy (ReEn) is an extension of the Shannon entropy with a tunable parameter α, allowing it to emphasise different aspects of a probability distribution.	α=2 and α=3	[41]

**Table 2:** Description of the entropy features used in this study together with the input parameters. Part 2/3.

Id	Description	Input Parameters	Reference
17	Permutation entropy (PeEn) is based on the analysis of the permutations of subsequences of size m in the time series on which this measure is applied.	m = 2	[42]
18,19,20, 21,22	Attention entropy (AttEn) is based on the concept of analysing the frequency distribution of the intervals between the key observations in a time-series instead of counting the frequency of all observations as in Shannon entropy. Attention entropy does not need any parameter to tune, it is robust to the time-series length, and requires only linear time to compute.	Natural Log	[36]
23	Distribution entropy exploits the information hidden in the state space by the estimation of the probability density of distances among vectors. In this context, this entropy measure addresses limitations of the sample entropy in stability and consistency would arise from the incomplete use of vector-to-vector distances information in the state space. The main hyper-parameters are the same of sample entropy with embedding parameter m and the delay τ.	m = 4, τ=1	[43]
24	Increment entropy is based on the increment information in a time series. Each increment is mapped onto a word of two letters, one corresponding to the sign and the other corresponding to the magnitude. Increment entropy is defined as the Shannon entropy of the words.	m = 3	[44]
25	Phase entropy is based on a quantification of a two-dimensional phase space. The entropy measure uses the second-order difference plot (SODP), a two-dimensional phase space, which provides a visual summary of the rate of variability in a time series. The distribution of scatter points in a SODP provides information about the dynamics of the underlying system. The phase entropy is estimated using the Shannon entropy of the weighted distribution in a coarse-grained SOD. Phase entropy is free of parameters		[45]

**Table 3:** Description of the entropy features used in this study together with the input parameters. Part 3/3.

Id	Description	Input Parameters	Reference
26,27,28	Slope entropy addresses one of the weaknesses of permutation entropy, which disregards the time series amplitude information. Slope entropy (SlopEn), addresses this flaw by keeping the symbolic representation of subsequences in the time series using a novel encoding method based on the slope generated by two consecutive data samples.	m = 4	[46]
29	Symbolic Dynamic entropy is another newly introduced entropy measure. It uses a conversion of the time series into the symbol time series (called symbolisation). The amplitude domain of the time series is partitioned into e cells. Each of the elements corresponds to a unique cell. On the symbol representation, the algorithm constructs the state transitions and compute the probability of state transitions on which the Shannon entropy is calculated.	m = 3	[47]
30, 31	Dispersion entropy is an improvement of the permutation entropy, where the mean value of amplitudes and differences between amplitude values are not considered in its definition. In dispersion entropy the initial values of the time series are mapped to c classes using the Normal Cumulative Distribution Function (NCDF)	m = 3, c = 3 and m = 3, c = 4	[48]
32, 33, 34, 35, 36, 37	The Gridded distribution entropy (GDE) is based on the application of the Poincaré plot, which is a return map that geometrically analyses the progression of a time-series. The algorithm applies a gridded Poincaré plot by coarse-graining the original graph created by the time series to generate the GDE, which is the Shannon entropy of the grid weight, i.e., the number of points in each grid	m = 3	[49]

**Table 4:** Description of the non-entropy features used in this study together with the input parameters.

Id	Description	Input Parameters	Reference
38	maximum value of the time series.	None	[50]
39	Skewness is the measure of asymmetry in a time series.	None	[50]
40	Kurtosis is the measure of tailedness or impulsiveness in a time series.	None	[50]
41	Burstiness is a statistical measure to evaluate how much a time series is characterised by short timeframes of intensive activity followed by long times of no or reduced activity.	None	[51]
42	Mean of a time series.	None	[50]
43	Variance measures the spread of data points around the mean over time, indicating volatility or dispersion.	None	[50]
44, 45, 46	Quantile is a value that divides the time series ordered data points into specific portions, showing where a certain percentage of observations fall.	Percentages equal to 0.5, 0.7 and 0.9	[52]
47, 48, 49	Auto mutual information with τ time lag at which to calculate the mutual information.	τ with values 1, 2, 3	[53]
50	The high low mu statistics is the ratio of the mean of the data that it is above the global mean compared to the mean of the data that is below the global mean.	none	[53]
51	The negative log-likelihood of data coming from a Gaussian distribution fits a Gaussian distribution to the data using the normfit function and return the negative log likehood of the data coming from a Gaussian distribution using the normlike function.	none	[53]
52, 53, 54	The range evolution measures how the time-series range changes across time captured with proportions.	proportion p equal to 1, 10 and 20.	[53]

**Table 5:** Numerical results and comparison with results on the eWine data set in the literature.

Method	Accuracy	Reference
Baseline BA (no IS)	79.99	This study
BA IS NB	83.20	This study
BA IS RF	84.97	This study
BA IS SVM	84.66	This study
ReliefF IS NB	83.62	This study
ReliefF IS RF	84.97	This study
ReliefF IS SVM	85.44	This study
IFS IS NB	82.88	This study
IFS IS RF	85.10	This study
IFS IS SVM	85.44	This study
MRMR IS NB	83.02	This study
MRMR IS RF	84.65	This study
MRMR IS SVM	84.14	This study
KNN	78.5	[15]
Supervised Time Series Forest	0.805	[9]
Supervised Time Series Forest with entropy features	0.811	[9]
Spiking Neural Networks	81.8	[19]
CNN+LSTM	81.56	[7]
CNN+Bi-LSTM	78.93	[7]
CNN+staked-LSTM	82.14	[7]
CNN and denoising	81.68	[18]
CNN-BiLSTM with Transfer Learning	83.85	[54]
LighMamba DL	87.1	[20]
CNN-BiLSTM-transformer	87.50	[8]
ADCNN-BiLSTM	89.06	[8]
ADCNN-BiLSTM-transformer	92.19	[8]

**Table 6:** Computing times.

Operation	Times in Seconds
Entropy features calculation (100 samples)	8 (0.08 s per sample)
Non-entropy features calculation (100 samples)	0.25 (0.0025 s per sample)
ReliefF	0.5
IFS	0.138
mRmR	0.3
Random Forest Training	12.3
Random Forest Inference	0.1
Naive Bayes Training	1.1
Naive Bayes Inference	0.3
Support Vector Machine Training	5.1
Support Vector Machine Inference	0.4

## Data Availability

The data is publicly available at https://github.com/ewine-project/UWB-LOS-NLOS-Data-Set. Last accessed 9 July 2026.
